# Annotation matters: the effect of structural gene annotation on orthology inference

**DOI:** 10.1093/bioinformatics/btaf365

**Published:** 2025-06-25

**Authors:** Silvia Prieto-Baños, Yannis Nevers, Adrian Altenhoff, Alex Warwick Vesztrocy, Christophe Dessimoz, Natasha M Glover

**Affiliations:** Department of Computational Biology, University of Lausanne, Lausanne, 1015, Switzerland; SIB Swiss Institute of Bioinformatics, Lausanne, 1015, Switzerland; Department of Computational Biology, University of Lausanne, Lausanne, 1015, Switzerland; SIB Swiss Institute of Bioinformatics, Lausanne, 1015, Switzerland; SIB Swiss Institute of Bioinformatics, Lausanne, 1015, Switzerland; Computer Science, ETH Zurich, Zurich, 8092, Switzerland; Department of Computational Biology, University of Lausanne, Lausanne, 1015, Switzerland; SIB Swiss Institute of Bioinformatics, Lausanne, 1015, Switzerland; Department of Computational Biology, University of Lausanne, Lausanne, 1015, Switzerland; SIB Swiss Institute of Bioinformatics, Lausanne, 1015, Switzerland; Department of Computational Biology, University of Lausanne, Lausanne, 1015, Switzerland; SIB Swiss Institute of Bioinformatics, Lausanne, 1015, Switzerland

## Abstract

**Motivation:**

*In silico* gene annotation, the process of identifying the genes present in a genome, remains a challenging task. As genome assemblies rapidly increase, the corresponding gene models and repertoires often fall short in quality. Despite advances in annotation methods, a lack of community standards means that most published gene annotations result from *ad hoc* pipelines. As a result, only a few species have nearly complete and accurate gene models. This annotation quality is thought to affect downstream analyses, including orthology inference, often the first step of comparative genomics studies.

**Results:**

We show that different annotation methods yield markedly distinct orthology inferences. We compared orthology assignments of gene models obtained by four prominent protein-coding gene model sources: the NCBI Eukaryotic Genome Annotation Pipeline, the Ensembl Gene Annotation System, the UniProt Reference Proteomes, and Augustus 3.4 (an *ab initio* pipeline). We observe significant discrepancies between sources, namely in the proportion of orthologous genes per genome, the completeness of Hierarchical Orthologous Groups, and the accuracy and recall of the predicted orthologs on a standard orthology benchmark.

## 1 Introduction

The genomics era has led to the accumulation of thousands of genome sequences across the Tree of Life, bolstered by ambitious projects such as the Earth BioGenome project or the European Reference Genome Atlas (Koepfli *et al.* 2015, Lewin *et al.* 2018, Rhie *et al.* 2021). More genomes will enhance comparative genomics thanks to improved representation of genomic diversity, providing insights into genome evolution and the relationship between genotype and phenotype (Feng *et al.* 2020, Blaxter *et al.* 2022). Accurate orthology inference underpins many comparative genomics analyses: it enables large-scale gene function prediction through annotation transfer from model organisms, supplies the homologous markers needed for phylogenomic reconstruction of species trees, and drives phylogenetic profiling and phylostratigraphy approaches by linking gene presence–absence patterns to phenotypes and dating gene-birth events across the tree of life (Glover *et al.* 2019, Linard *et al.* 2021, Langschied *et al.* 2024). The cornerstone of these studies lies in the accurate identification of orthologous sequences, given that orthology (and paralogy) inference is typically their prerequisite (Gabaldón and Koonin 2013, Kirilenko *et al.* 2023).

The most common input for orthology inference pipelines is the sequences of all protein-coding genes annotated in the genomes of interest. These genes are computationally inferred by identifying the genomic coordinates that code for proteins and often are subsequently translated to amino acid sequences *in silico*. Here, we refer to this process as gene annotation, to protein-coding genes as genes, and the entire translated protein-coding gene repertoire as the proteome.

Finding the genes in a genome is not a trivial task, especially in eukaryotic species in which most of the sequence is noncoding and the coding regions are separated by intronic sequences (Salzberg 2019). Gene identification is commonly performed with a mix of three types of evidence: *ab initio* gene prediction, homology-based inference, and transcriptomic data (Yandell and Ence 2012). However, the pipelines and evidence used for annotation vary greatly, leading to heterogeneity in the annotations available in public databases, even within the same species. These annotation challenges can cause complications, mistakes, and false discoveries in downstream analyses (Bányai and Patthy 2016, Zile *et al.* 2020, Weisman *et al.* 2022).

Orthology inference is generally the first step in comparative genomics analyses, and studies assessing orthology acknowledge that gene annotation may influence orthology results. Notably, Trachana *et al.* (2011) showed that the proteome’s source is a significant confounder in orthology methods assessment. Furthermore, Weisman *et al.* (2022) found that using heterogeneous genome annotations, a common approach in comparative genomics studies, spuriously inflated the number of lineage-specific genes, and suggested a similar effect on gene losses. While there are tools to evaluate genome and proteome quality (e.g. BUSCO (Manni *et al.* 2021) and OMArk (Nevers *et al.* 2025)), the influence of annotation methods on orthology remains underexplored.

We assessed the influence of different gene annotation sources on orthology across 20 Chordata genomes. Specifically, we evaluated proteomes generated by: (i) the Ensembl gene annotation system (Aken *et al.* 2016, Cunningham *et al.* 2022), (ii) the NCBI eukaryotic genome annotation pipeline (Thibaud-Nissen *et al.* 2016, Goldfarb *et al.* 2025), (iii) UniProt’s Reference Proteomes, and (iv) the *ab initio* method Augustus (Stanke and Waack 2003, Hoff and Stanke 2019). The first three sources use pipelines that incorporate several evidence types, including *ab initio* predictions, transcriptomic data, and homology, with manual curation in some cases. Augustus is one of the most commonly used *ab initio* methods, and although it can integrate evidence into its gene predictions, we used it exclusively in its *ab initio* capacity to generate gene models without additional evidence. This approach served as a baseline to contrast with the more comprehensive annotation pipelines.

We inferred orthology on the proteomes annotated by the four methods and observed that the annotation method significantly impacts orthology assignment in terms of accuracy, recall, the proportion of orthologous genes and the quality of the gene families. Notably, disparities observed among methods were evident not only between *ab initio* and the other methods, but also among established annotation resources. We further show that the difference in orthology results is related to differences in protein length for each method. Our findings call for awareness of the choice of annotation pipelines for orthology inference and downstream analyses and highlight the need for community standards in genome annotation.

## 2 Materials and methods

### 2.1 Selection of species, assemblies, and proteome annotations

**Figure 1.**
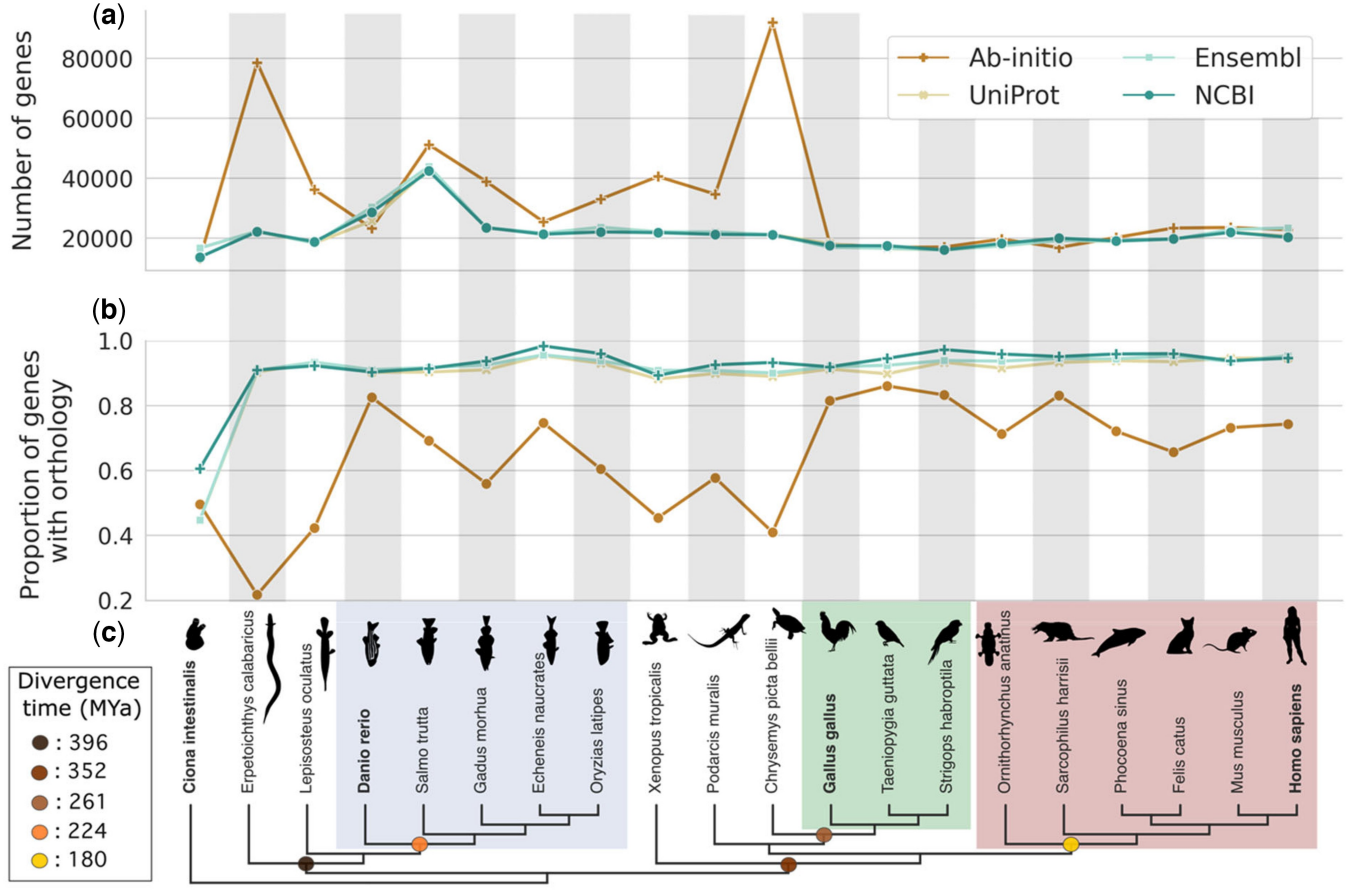
Difference in predicted number of genes and their proportion with orthologs across annotation methods in the 20 chordata species. (a) Number of protein-coding genes inferred by each annotation method. (b) Proportion of genes with at least one ortholog for each species. (c) Cladogram of the species used in the analyses. Colored circles indicate the median estimated divergence time at the specified split by TimeTree5. Rectangles highlight different clades (blue: Teleostei, red: Aves, green: Mammalia) and species in bold are those which parameters were used in *ab initio* predictions (*Ciona intestinalis, Danio rerio, Gallus Gallus*, and *Homo sapiens*). Species illustrations were obtained from phylopic. (www.phylopic.org). Illustration of Chrysemys picta by B. O'Meara, Ciona intestinalis and Lepisosteus oculatus by C. Schomburg, Danio rerio by J.Warner, Echeneis naucrates by J. Heaven, Erpetoichthys calabaricus by G. Dera, Felis silvestris catus by SJS, Gadus morhua by A. Hahn, Gallus gallus by S. Traver, Homo sapiens by T.M. Keesey, M. musculus by K. S. Jaron, Ornithorhyncus anatinus by G. Dera, Oryzias latipes by seung9park, Phocoena sinus by C. huh (https://creativecommons.org/licenses/by-sa/3.0/), Podarcis erhardii by A. Slavenko, S. trutta by C. Cano-Barbacil, Strigops habroptila by M. Ramirez, Taeniopygia guttata by A. Wilson and Xenopus tropicalis by S. Werning (https://creativecommons.org/licenses/by/3.0/).

We selected 20 phylogenetically diverse Chordata species with chromosome-level assemblies and publicly available proteomes annotated by Ensembl and NCBI (Table S1, available as supplementary data at *Bioinformatics* online; Fig. 1). Using NCBI and the World Register of Marine Species taxonomies as a guide, we included the most representative species from major clades, favoring those with the most complete NCBI and Ensembl proteomes, as determined using OMArk (Nevers *et al.* 2025). The scope of our phylogeny was confined to Chordata primarily due to the availability of Ensembl-annotated genomes being limited to this phylum (Benjamin Moore, personal communication 2022).

To maintain consistency across annotation methods, we used identical assembly versions for all 20 genomes. We used the “primary” assemblies for Augustus gene prediction (see Supplementary Material, available as supplementary data at *Bioinformatics* online, for further information on primary versus top-level assemblies).

To obtain the *ab initio* annotations, we ran Augustus v3.4 (Stanke and Waack 2003, Hoff and Stanke 2019) on the soft-masked assemblies, sourced from Ensembl. This soft-masking ensures that Augustus recognizes the regions corresponding to repetitive elements. Specific genomes for training Augustus parameters were available for four species. For the remaining 16 species, we selected the evolutionarily closest species available (Table S1, available as supplementary data at *Bioinformatics* online). Divergence times between species and their corresponding parameters species were obtained using median time estimates from the TimeTree 5 project (Kumar *et al.* 2022).

We sourced proteomes for each species from Ensembl, NCBI RefSeq, and UniProt Reference Proteomes, all annotated on the same assembly versions. NCBI RefSeq and Ensembl use their own pipelines: the NCBI Eukaryotic Genome annotation pipeline and the Ensembl gene annotation system (Aken *et al.* 2016, Thibaud-Nissen *et al.* 2016, Cunningham *et al.* 2022, Goldfarb *et al.* 2025). Conversely, UniProt proteomes come from translations of INSDC genome sequences or from externally annotated genomes (UniProt Consortium 2023). In our case, all UniProt proteome sets used in this study originally came from Ensembl except *Xenopus tropicalis*, which was imported from NCBI RefSeq.

To test for significant differences in the resulting total proteins number between methods, the Wilcoxon signed-rank test was performed using the “stats” module from the *scipy* v 1.11.1 library (Virtanen *et al.* 2020).

To compare the genome annotations from different methods, we used Gffcompare v 0.12.6 (Pertea and Pertea 2020) using custom versions of the gff files provided by the annotation sources (NCBI RefSeq and Ensembl) which include only protein-coding genes. In the case of Augustus, only protein-coding genes are returned in the output gff. To our knowledge, UniProt does not provide a gff with the gene features of their reference proteomes. We ran the comparisons twice for each possible method combination, with a different method considered as reference and query each time. We extracted the genes that had at least one complete match (“=”) for one of their transcripts from the Gffcompare’s output “.tracking” files. We computed the Jaccard index for each set comparison. In some cases, the number of genes with matches was not symmetric between the query and the reference (e.g. where one reference matched two query genes), so we included them as a one-to-many relationship and considered the total number of genes involved. Finally, we averaged the two Jaccard indexes obtained for the two method pair comparisons for each species. To test for significant differences between the final average Jaccard indexes, we performed the Wilcoxon signed-rank test, again using the “stats” module from the *scipy* v 1.11.1 library.

### 2.2 Orthology inference

To infer orthologs, we ran OMA Standalone v2.5 (Altenhoff *et al.* 2019) four times, once for each annotation method’s resulting proteomes.

OMA was run with default parameters. The species tree topology was obtained from the NCBI taxonomy and used as input for OMA to build Hierarchical Orthologous Groups (HOGs). For genes encoding alternative splicing isoforms, OMA identifies the most evolutionarily conserved isoform (hereafter referred to as “canonical isoform”) when executed with “.splice” files to specify the isoform identifiers for each gene (Altenhoff *et al.* 2021).

We additionally ran OrthoFinder (Emms and Kelly 2019) to assess the effect of the orthology inference method on the Generalized Species Tree Benchmark results (see Supplementary Methods, available as supplementary data at *Bioinformatics* online). To compare OMA and OrthoFinder and to evaluate the effect of isoform choice on orthology, we ran OMA again, but using only the longest isoforms, and OrthoFinder, but only using the most conserved isoforms that OMA selects. This granted a fair comparison between OMA and OrthoFinder, as OrthoFinder chooses the longest isoforms by default (see Supplementary Methods, available as supplementary data at *Bioinformatics* online).

### 2.3 Orthology assessment

To evaluate the quality of the orthology inference results from each annotation source, we utilized various metrics, described below.

For each genome, we calculated the proportion of proteins inferred to have at least one ortholog using the “Pairwise Orthologs” output from OMA Standalone. We derived this proportion by dividing the number of proteins with an ortholog by the total number of proteins, only including canonical isoforms. Statistical comparisons between methods were performed using the Wilcoxon signed-rank test (*scipy* v 1.11.1). Furthermore, we evaluated the potential of genes without orthology to be lineage-specific genes by comparing their total count to estimated numbers of lineage-specific genes in each species (see Supplementary Methods, available as supplementary data at *Bioinformatics* online). To assess the overall quality of gene families (referred to as HOGs within the OMA framework) across different annotation methods, we used the following metrics: (i) the total number of HOGs generated; (ii) HOG size distribution, i.e. the number of genes per HOG; (iii) HOG completeness score, calculated as the number of species in a HOG divided by the total number of species in the relevant clade (Altenhoff *et al.* 2023); and (iv) number and distribution of HOGs with two or 20 genes. Statistical comparisons between annotation methods were performed using Welch’s ANOVA and the Games–Howell *post hoc* test (*pingouin* v 0.5.3). The HOG data were obtained from the output of OMA Standalone, namely “PhyleticProfileHOGs.txt” and the “HierarchicalGroups.orthoxml” files.

We adapted the Generalized Species Tree Discordance benchmark (Altenhoff *et al.* 2016, Nevers *et al.* 2022) for our analysis. This benchmark measures the accuracy and recall of orthology inference methods, based on the congruence between gene tree topologies and the species tree. This is premised on the notion that two genes are orthologous if they originated from a speciation event, and therefore, the topology of a gene tree constructed only with orthologs should equal that of the species tree. Accuracy is quantified using the average Robinson–Foulds (RF) distance between the gene trees and the species tree. The RF distance measures the dissimilarity between two phylogenetic trees by counting the partitions present in one tree but not in the other, scaled to a maximum value of 1. Recall is assessed as the number of gene trees that were successfully constructed from randomly selected protein sequences (i.e. the number of randomly selected genes which had orthologs in 10 out of the 20 included species) out of 50 000 trials. Both OMA and OrthoFinder outputs were used for the Generalized Species Tree Discordance orthology benchmark.

### 2.4 Protein length

To address annotations characteristics influencing orthology results, we examined the length of each annotation’s canonical isoforms. We obtained each species’ median protein length and correlated them with the proportion of genes with orthologs (Section 2.3) using Pearson’s correlation (*scipy* v 1.11.1). Secondly, we obtained the lengths for each annotations’ canonical proteins with orthologs and singletons (Section 2.3) and tested for differences between annotation methods using Welch’s ANOVA and the Games–Howell *post hoc* test (*pingouin* v 0.5.3). Finally, we compared all the species length distributions with the two-sample Kolmogorov–Smirnov test for goodness of fit (*stats* module, *scipy* v 1.11.1).

### 2.5 BUSCO and OMArk

We ran BUSCO v 5.7.1 and OMArk v 0.3.0 over all proteomes to investigate the relationship between annotation quality and the orthology inference results. We used the automatically detected clade-specific BUSCO datasets to evaluate the gene content.

## 3 Results

We used four annotation sources—the NCBI Eukaryotic Genome Annotation Pipeline, the Ensembl Gene Annotation System, the UniProt Reference Proteomes, and Augustus 3.4 (*ab initio*)—to evaluate the effect of annotation on orthology inference across 20 species.

### 3.1 Protein-coding gene assessment

**Figure 2.**
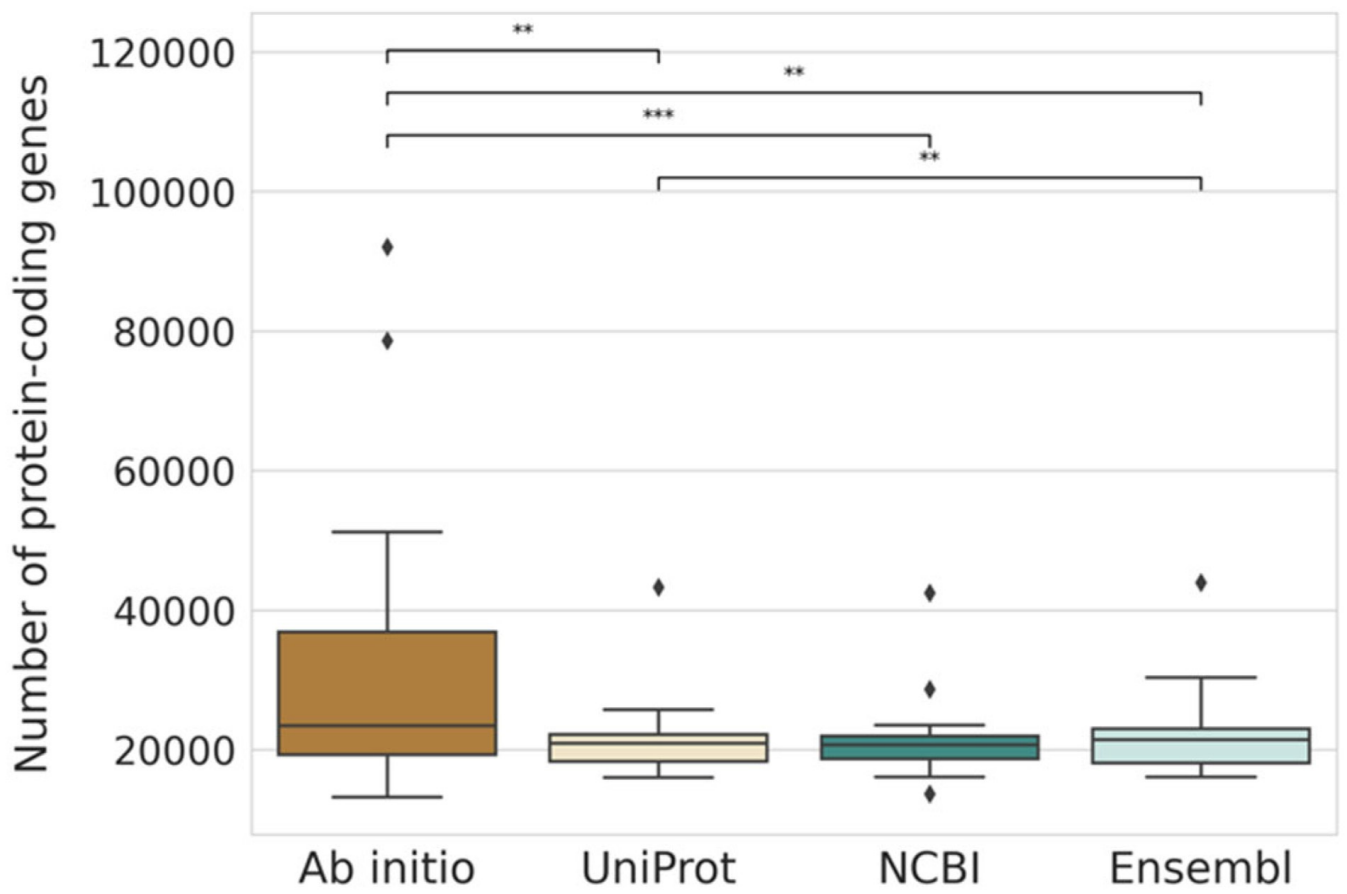
Number of genes predicted for all species per method. Asterisks represent *P*-values from the Wilcoxon test (***<.001, **<.01, *<.05).

We first assessed the difference in the number of genes predicted by each annotation (Fig. 1a). Our findings reveal that across species, *ab initio* generally predicted a substantially and significantly higher number of genes compared to Ensembl, UniProt, and NCBI (Fig. 2, Tables S2 and S3, available as supplementary data at *Bioinformatics* online).

The median number of genes predicted for UniProt, Ensembl, and NCBI were similar, although UniProt had slightly but significantly fewer genes than Ensembl (Fig. 2, Wilcoxon *P* = .002). The higher variability in the number of genes predicted by Augustus (*ab initio*) is likely dependent on the species used for training (Fig. S2, available as supplementary data at *Bioinformatics* online).

**Figure 3.**
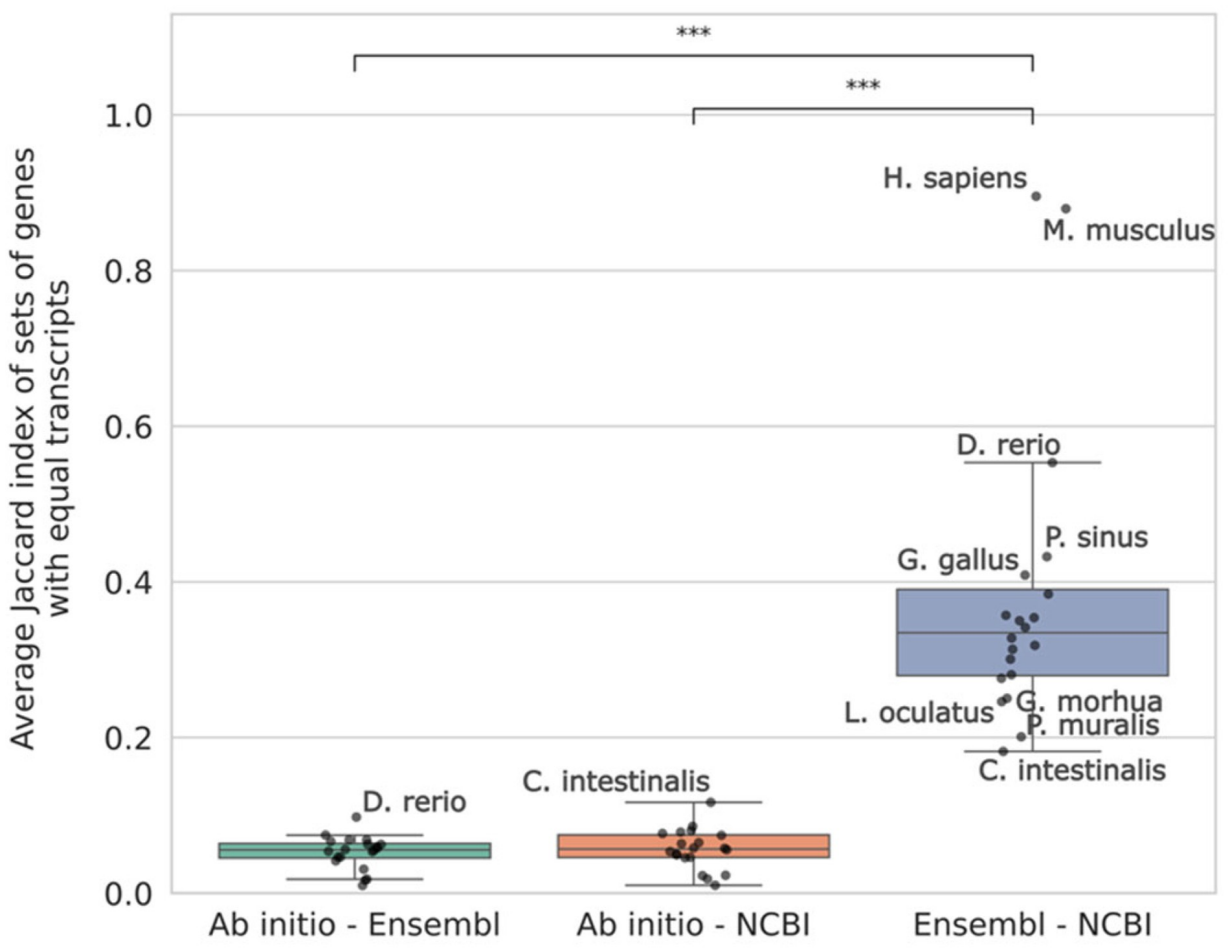
Pairwise similarity of gene models, quantified by the Jaccard index for each annotation method comparison. For each method pair, we ran GffCompare twice (swapping reference and query) and report the mean of the two resulting Jaccard indexes. Asterisks represent *P*-values from the Wilcoxon test (***<.001, **<.01, *<.05).

Next, we checked the agreement between gene models from different methods using GffCompare (Pertea and Pertea 2020). UniProt proteomes were not included, as their features are not available online. We consider that two gene models “agree” if at least one of their transcripts have a complete match. This implies all introns and most exons being identical, allowing for some flexibility at the external boundaries of the terminal exons. We computed the agreement between Ensembl, NCBI, and *ab initio* for each species. All method pair combinations were computed twice, exchanging the method considered as query and reference. We report the average Jaccard indexes (Ji) of both computations (Fig. 3), which in all cases differed by less than 0.01 (Table S5, available as supplementary data at *Bioinformatics* online).

We recover few identical gene models between annotation methods, however the Ensembl and NCBI gene sets are significantly more similar (Fig. 3, median Ji = 0.33) than those of *ab initio* and either Ensembl (median Ji = 0.06) or NCBI (median Ji = 0.06). On the other hand, *ab initio* gene models did not agree particularly better with any of the other methods (Wilcoxon *P* = .31). The agreement varies depending on the species considered, especially when comparing Ensembl and NCBI, with *Homo sapiens* and *Mus musculus* showing substantial higher agreement (Ji = 0.89 and Ji = 0.88) than the rest of the species, likely reflecting the focus on these species of curation efforts. *Ciona intestinalis* showed the lowest (Ji = 0.18) agreement between NCBI and Ensembl. In the *ab initio—*Ensembl and *ab initio—*NCBI comparisons, the Jaccard indexes for each species do not vary as much, but *Danio rerio* and *C. intestinalis* have the highest similarities, respectively (Fig. 3).

We expect UniProt gene models’ agreement to resemble the Ensembl results, as all UniProt proteomes in our dataset except *Xenopus tropicalis* were based on Ensembl annotations.

### 3.2 Orthology results assessment

We evaluated the orthology results for the four annotation sets using three main approaches: the proportion of genes with orthologs, assessment of HOGs and an adapted version of a benchmark typically used to evaluate orthology inference methods.

#### 3.2.1 Proportion of orthologous genes

We first assessed the difference in orthology inference by evaluating the proportion of predicted genes inferred to have an ortholog. Barring true lineage-specific genes, accurate gene models are expected to render proteins that are orthologous to proteins from relatively close species.

**Figure 4.**
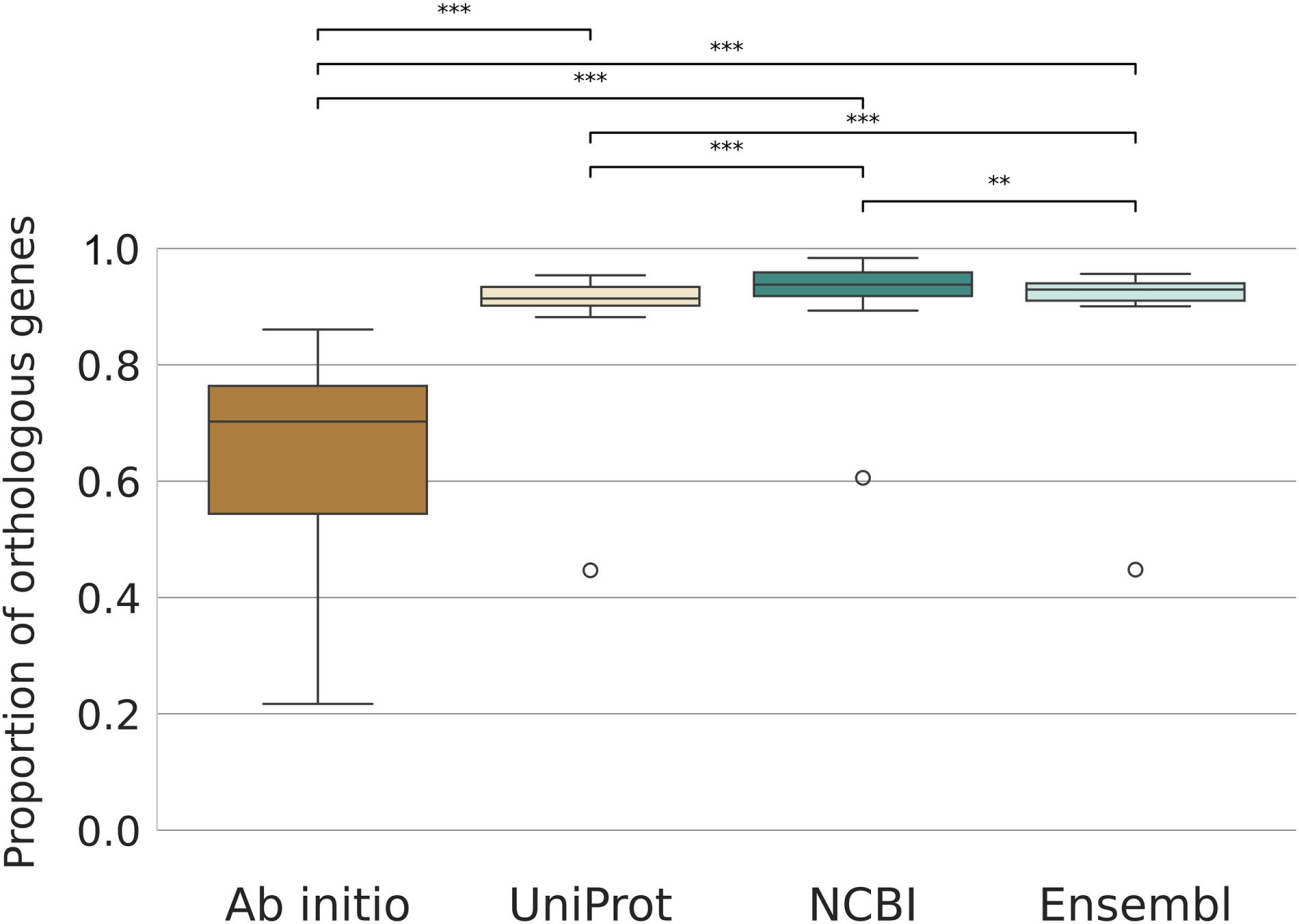
Proportion of genes that are part of an OMA-inferred orthologous pair, by method. Asterisks represent the *P*-values of the Wilcoxon test (***<.001, **<.01, *<.05).

We calculated the proportion of genes involved in at least one orthologous pair for each species (Fig. 1b). The median proportion of genes with orthology across species was 0.70 for *ab initio*, 0.91 for UniProt, 0.93 for Ensembl, and 0.94 for NCBI (Table S2, available as supplementary data at *Bioinformatics* online, Fig. 4). The proportions of orthologous genes were significantly different between every method, although *ab initio* was markedly different, with the lowest proportion of orthologs among all methods (Fig. 4; Tables S2 and S6, available as supplementary data at *Bioinformatics* online).

For the majority of the species (19/20), the *ab initio* gene set shows the lowest proportion of genes with an ortholog, with *C. intestinalis* being the exception (Fig. 1b). In most of these cases, the number of singletons exceeds the expectations based on estimates of de novo gene emergence (Fig. S4, available as supplementary data at *Bioinformatics* online), suggesting erroneous non-orthologous genes rather than true lineage-specific genes. *Ab initio* predictions having the lowest proportion of genes with an ortholog mirrors the differences in protein number discussed earlier, with a negative correlation between the number of proteins predicted by *ab initio* and the proportion of genes with an ortholog (Pearson’s *r* = −0.74, *P* < .001; Fig. S2, available as supplementary data at *Bioinformatics* online). Furthermore, there is also a negative correlation between the proportion of genes with an ortholog in Augustus and the divergence time of the species being annotated relative to the species’ parameters used (Pearson’s *r* = −0.72, *P* < .001, Fig. S3, available as supplementary data at *Bioinformatics* online). These results suggest that the inflated gene predictions by Augustus often result in erroneous non-orthologous genes, especially for species evolutionarily distant from the ones in the training set.

Our findings show the impact of annotation source on the resulting number of genes related by orthology, highlighting not only the large disparity introduced by insufficiently trained *ab initio* methods but also the moderate differences among other annotation sources.

#### 3.2.2 Assessment of HOGs

HOGs provide a structured framework for evaluating orthology assignment across species, as they are defined as sets of homologs that descended from a common ancestral gene at a specified taxonomic level. In this way, HOGs represent gene families.

**Figure 5.**
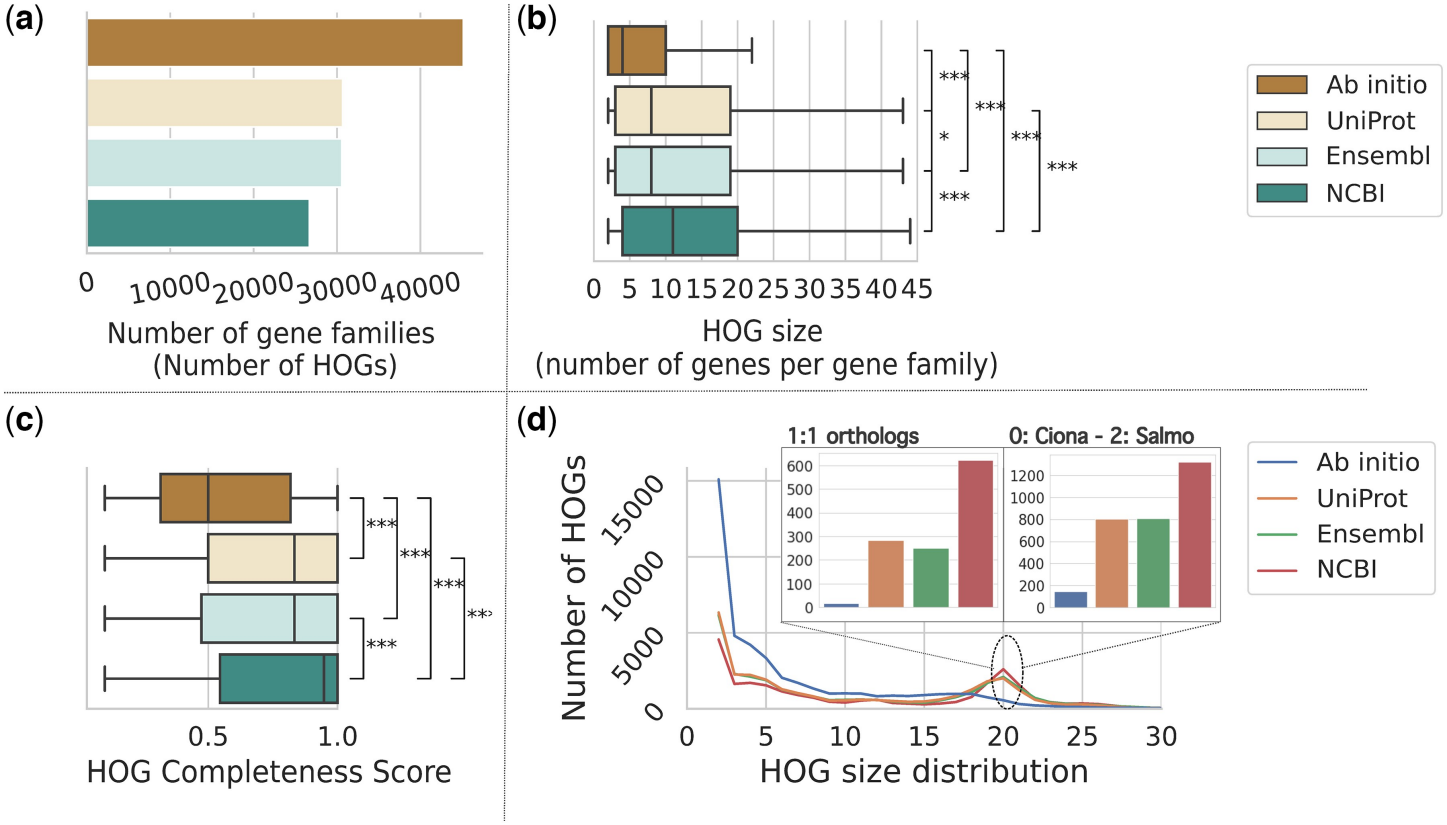
HOG metrics using OMA orthology. (a) Number of rootHOGs inferred by OMA over all clades; (b) RootHOG size distribution (number of genes per HOG); asterisks represent the *P*-values of the Games–Howell test (***<.001, **<.01, *<.05); outliers are not shown (see Fig. S4, available as supplementary data at *Bioinformatics* online, for outliers); (c) RootHOGs Completeness score distribution, i.e. number of species present in the HOG out of the total number of species in the clade where the HOG originated; asterisks represent the *P*-values of the Games–Howell test (***<.001, **<.01, *<.05); (d) RootHOGs with <30 genes size distribution. Zoom in of HOGs with 20 1:1 orthologous genes (one gene per species—single-copy genes) (left) and HOGs with two genes in *Salmo trutta*, no genes in *Ciona intestinalis* and one gene in the rest of species (right).

For a given gene family, the “rootHOG” is the largest group of homologous genes which OMA infers, and it comprises all its nested, descendant HOGs. The taxonomic level of the rootHOG indicates the origin of the ancestral gene from which all the members of the HOG descend. HOGs with few genes relate proteins in only a few species, whereas bigger HOGs represent deeper orthology relationships and/or include paralogous genes (those that emerged from duplication events). In OMA, challenges from recognizing homology may result in the inability to group HOGs together in one rootHOG, resulting in “split” HOGs and a higher overall number. Therefore, better quality proteins are expected to render fewer, bigger, and phylogenetically deeper rootHOGs. Our analysis showed that the *ab initio* proteomes set renders the most rootHOGs (45 153), followed by UniProt (30 658), Ensembl (30 576), and NCBI (26 678) (Fig. 5a, Table S7, available as supplementary data at *Bioinformatics* online).

The size of the HOGs depends on the number of species in the dataset and the evolutionary events a gene family has been subjected to. In our dataset, we expect to find a substantial number of HOGs with 20 genes, given that we include 20 species in the analysis. However, teleosts underwent a whole genome duplication event ∼270 MYA (Qi *et al.* 2024), and *Salmo trutta* underwent a second one ∼80 MYA (Lien *et al.* 2016). Such events are typically followed by loss of most duplicated genes, with ∼80% of the duplicated genes from the teleost-specific WGD lost within 60 MY (Inoue *et al.* 2015) and over 60% of the salmon-specific WGD duplicates lost (Gundappa *et al.* 2022). Therefore, we expect a substantial number of HOGs with 20 single-copy genes, one per species, and of HOGs with two copies in *S. trutta* and a missing gene in one species, namely *C. intestinalis*, as it’s the phylogenetically most distant species within our set.

We observed variations in HOG size across orthology results for different annotation methods (Welch’s ANOVA *P* = .0, Fig. 4b, Table S7, available as supplementary data at *Bioinformatics* online). HOGs with two genes were notably prevalent (Fig. 5d), comprising up to 33.4% of *ab initio*’s HOGs compared to 17.1%–20.7% for the other methods, indicating potential over-splitting of the HOGs due to failure to detect homology. Ensembl and UniProt had similar percentages of two-member HOGs (20.2% and 20.7%, respectively), whereas NCBI had the lowest at 17.1%. Furthermore, orthology assignment on the NCBI dataset excelled at accurately capturing HOGs with strictly 20 genes, one from each species: 621 1:1 HOGs, compared to 283 for UniProt, and 250 for Ensembl (Fig. 4d). In contrast, *ab initio* lagged significantly, with only 19 such 1:1 HOGs. Finally, NCBI also recovered the most 20-member HOGs with no genes in *C. intestinalis* and two copies in *S. trutta*: 1319 HOGs, compared to 1087 for UniProt, 1059 for Ensembl, and 163 for *ab initio* (Fig. 5d).

These analyses show that orthology inference on the NCBI annotations resulted in more accurate and complete rootHOGs defined at the Chordata level.

HOG Completeness (Fig. 5c), another metric to assess HOG quality (Altenhoff *et al.* 2023), measures the proportion of species represented by at least one gene in a HOG relative to the total number of species in the clade the HOG is defined at. Given the relatively low propensity for gene losses in diploid genomes (Albalat and Cañestro 2016), high Completeness scores are expected. Our analysis revealed NCBI’s HOGs had the highest average Completeness score at 0.77, suggesting a more comprehensive coverage. UniProt and Ensembl’s HOGs closely followed NCBI with an average score of 0.71, indicating slightly less but still substantial completeness, whereas *ab initio* had the lowest at 0.56.

Across these HOG quality metrics, a clear and consistent trend emerged. The *ab initio* approach produced generally less complete and more fragmented HOGs compared to other methods. Conversely, NCBI's annotations resulted in the most comprehensive and cohesive HOGs, indicating better orthology inference. Ensembl and UniProt’s performance positioned between *ab initio* and NCBI, leaning closer to the latter.

#### 3.2.3 Generalized Species Tree Discordance benchmark

We adapted the Generalized Species Tree Discordance orthology benchmark (Altenhoff *et al.* 2016) to evaluate the orthology from different annotation sources. This benchmark computes accuracy and recall, where recall is a proxy of the proportion of orthologous groups which include at least 10 species, and the error is the average scaled RF distance between the reconstructed gene trees and the species tree.

**Figure 6.**
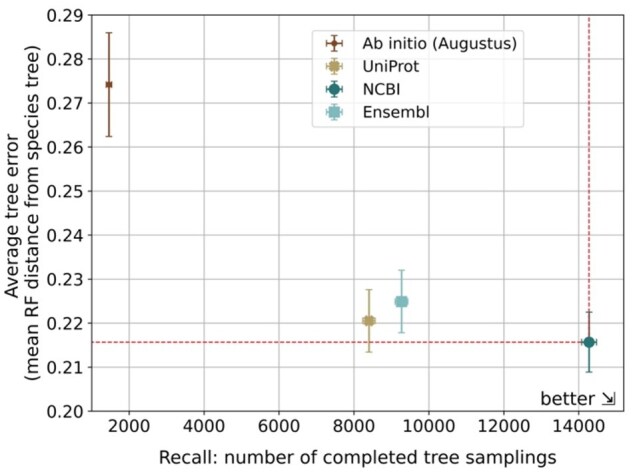
Generalized Species Tree Discordance Benchmark results for OMA. Average tree error provides an estimate of inverse accuracy, and the recall consists of the number of successful tree samples out of 50 000 trials.

We ran the benchmark for each annotation’s orthology results computed using OMA and OrthoFinder to compare the annotation’s performance using two different orthology inference methods. For OMA results, NCBI achieved the highest recall (completed gene trees: 14 277) and accuracy (mean RF distance: 0.216) (Fig. 6). In contrast, *ab initio* shows the lowest recall (trees: 1462) and accuracy (mean RF distance: 0.274). Ensembl and UniProt’s recall and accuracy lied in between *ab initio* and NCBI. For OrthoFinder, the results were comparable to those obtained with OMA (Fig. S6, available as supplementary data at *Bioinformatics* online). Interestingly, there was a difference between OMA and OrthoFinder in the recall of Ensembl and UniProt datasets. For OMA, Ensembl had a higher recall (9271) than UniProt (8402); for OrthoFinder, UniProt had a higher recall (16 954) than Ensembl (14 567) (Fig. S6, available as supplementary data at *Bioinformatics* online). This is likely due to the different approach in handling isoforms that OMA and OrthoFinder follow: OrthoFinder selects the longest isoform, whereas OMA selects the most evolutionarily conserved isoform. Using the longest isoforms in OMA, the relative rankings between methods were identical between OrthoFinder and OMA. Moreover, using the most evolutionarily conserved isoforms in OrthoFinder, Ensembl, and UniProt achieved nearly identical recall values (Fig. S7 and Table S8, available as supplementary data at *Bioinformatics* online). These results indicate that the combination of annotation method, isoform selection strategy, and orthology inference algorithm all have an impact on orthology calls. Nevertheless, the benchmark results confirm the pattern observed among annotation methods in previous metrics, indicating that the results are due to the annotation sources rather than the orthology inference method.

### 3.3 Protein length comparison

**Figure 7.**
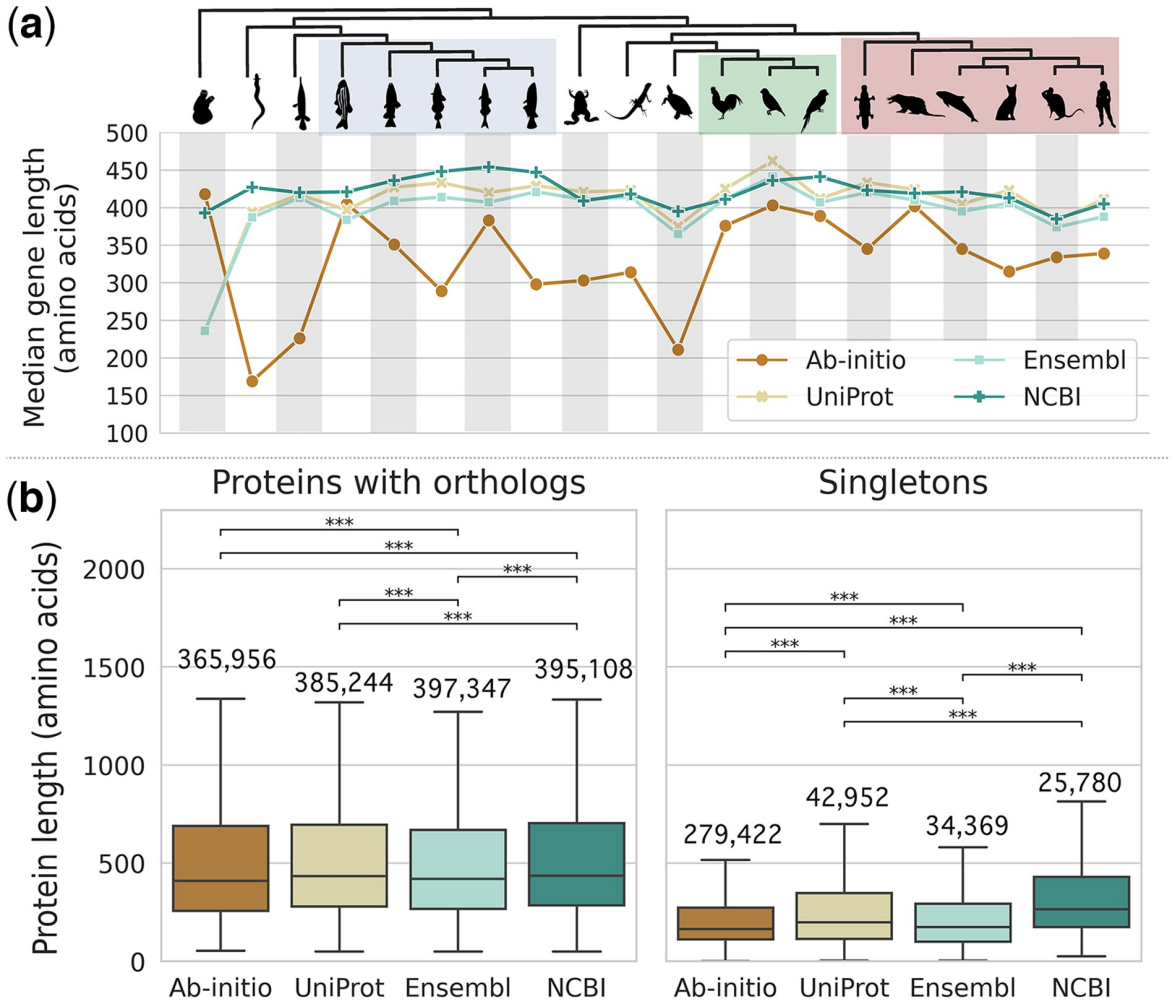
Protein length. (a) Median protein lengths across species (indicated by the cladogram on top) for each annotation method. (b) Protein length distributions of proteins with orthology (left) and of proteins not orthologous to any other protein (right); the number on top of each box indicates the total number of proteins assigned to each group; asterisks represent the *P*-values of the Games–Howell test (***<.001, **<.01, *<.05); outliers are not shown. (www.phylopic.org). Illustration of Chrysemys picta by B. O'Meara, Ciona intestinalis and Lepisosteus oculatus by C. Schomburg, Danio rerio by J.Warner, Echeneis naucrates by J. Heaven, Erpetoichthys calabaricus by G. Dera, Felis silvestris catus by SJS, Gadus morhua by A. Hahn, Gallus gallus by S. Traver, Homo sapiens by T.M. Keesey, M. musculus by K. S. Jaron, Ornithorhyncus anatinus by G. Dera, Oryzias latipes by seung9park, Phocoena sinus by C. huh (https://creativecommons.org/licenses/by-sa/3.0/), Podarcis erhardii by A. Slavenko, S. trutta by C. Cano-Barbacil, Strigops habroptila by M. Ramirez, Taeniopygia guttata by A. Wilson and Xenopus tropicalis by S. Werning (https://creativecommons.org/licenses/by/3.0/)

Orthology inference is commonly impacted by the sequences’ length, as it usually relies on sequence alignment. *Ab initio* exhibited the lowest median protein length, followed by Ensembl, UniProt, and NCBI (Fig. 7a, Table S7, available as supplementary data at *Bioinformatics* online; 286, 396, 409, and 422 amino acids, respectively).

Moreover, good quality gene repertoires are expected to render uniform protein length distributions across the different species (Nevers *et al.* 2023). We assessed each annotation method’s results’ conformity to this expectation and examined where genes with orthologs fall within this distribution in comparison to singletons. All methods show positively skewed length distributions (Fig. S8, available as supplementary data at *Bioinformatics* online), as expected (Nevers *et al.* 2023). We used the Kolmogorov–Smirnov (KS) statistic to quantify the distance between two distributions—0 indicates identical distributions, while 1 indicates maximum dissimilarity. The variability between the species’ length distributions is the highest within *ab initio* (average KS statistic = 0.13), then UniProt and Ensembl (avg. KS = 0.07 and 0.06, respectively) and finally NCBI (avg. KS = 0.05) (Figs. S9 and S10, available as supplementary data at *Bioinformatics* online). Notably, *C. intestinalis*, *Chrysemys picta bellii*, and *Erpetoichthys calabaricus* had the most variable protein lengths between different annotations (Figs. S10 and S11, available as supplementary data at *Bioinformatics* online).

The comparison of proteins with at least one ortholog to singletons (proteins without orthologs) revealed that proteins with orthologs are consistently longer across all methods (Fig. 7b). In contrast, singletons tend to be shorter, suggesting that some singletons might be due to fragmented, incomplete, or spurious gene models rather than true unique genes. Furthermore, the median protein length for each species is strongly correlated to the proportion of genes with an ortholog (Pearson’s *r* = 0.88, *P* < .001). Notably, *ab initio* produced not only the highest number of singletons, but also the shortest (median length 164 aa; Fig 7b). NCBI, on the other hand, showed the fewest and longest singletons (median protein length 264 aa; Fig 7b), indicating a potentially higher proteome quality.

The results of the protein length analysis reflect the results from the orthology inference assessments in previous sections; NCBI exhibited the longest proteins (including both singletons and proteins with orthologs) with the least variation in length distribution. Conversely, *ab initio*’s shorter proteins and more singletons indicate a propensity for over-predicting putatively erroneous genes, potentially misleading downstream comparative genomics analyses.

### 3.4 BUSCO and OMArk

The evaluation of protein-coding gene annotations can significantly benefit from comparative tools like OMArk and BUSCO (Manni *et al.* 2021, Nevers *et al.* 2025), which benchmark genomes against clade-conserved gene families. We ran these tools on the proteomes to pinpoint important metrics for orthology quality. Both tools evaluate some similar metrics, such as proteome Completeness, namely in terms of the proportion of “Missing” genes, i.e. those present in most closely related species but not in the assessed gene repertoire. “Complete” and “single-copy” genes in BUSCO or “Single” genes in OMArk, are the genes present in most closely related species that can be found in a single copy in the evaluated proteome. OMArk additionally evaluates the taxonomic consistency of proteomes, where “Taxonomically Consistent” genes are those genes from the query proteome that match to the lineage’s known gene families. “Taxonomically Inconsistent” genes match to gene families outside the lineage. “Unknown” genes do not match any gene family in the OMA database—meaning, there was no detected homology.

Both BUSCO’s and OMArk’s outputs show a clear difference in annotation quality between our baseline *ab initio* proteomes and the other three methods, reporting many more missing and fragmented genes (Figs. S12 and S13, available as supplementary data at *Bioinformatics* online). The median number of missing genes for *ab initio* across all species was 14.10% based on the OMArk results and 16.35% based on the BUSCO results (Fig. S14, available as supplementary data at *Bioinformatics* online). NCBI shows slightly less missing genes (2.45% OMArk, 1.65% BUSCO) than UniProt (2.61% OMArk, 4.15% BUSCO) and Ensembl (2.66% OMArk, 4.45% BUSCO). Moreover, in the OMArk output for the *ab initio* annotations, we also find a higher percentage of “Unknown” genes, i.e. those query genes with no detected homology (Fig. S15, available as supplementary data at *Bioinformatics* online). The differences between UniProt, Ensembl, and NCBI are more subtle (Fig. S15, available as supplementary data at *Bioinformatics* online). The percentage of Taxonomically consistent genes in *ab initio* is also the lowest (median = 84.21%, Fig. S16, available as supplementary data at *Bioinformatics* online), with the values for Ensembl, UniProt, and NCBI being more homogeneous, this time NCBI ranking the lowest (97.63%, 97.76%, and 96.56%, respectively).

Considering OMArk results, we find that for baseline annotations (*ab initio*), the percentage of Unknown genes and the percentage of Taxonomically consistent genes are the metrics most correlated to the percentage of genes with an ortholog in our results (Pearsons’s *r* = −0.90 and *r* = 0.92, respectively, Fig. S17, available as supplementary data at *Bioinformatics* online). For the three other methods, we find that not only are the aforementioned metrics highly correlated to the percentage of genes with orthologs (Pearson’s *r* anging from 0.87 to 0.99; Figs. S18 and S19, available as supplementary data at *Bioinformatics* online), but also the percentage of missing genes (Pearson’s: Ensembl *r *= 0.86, UniProt *r *= 0.87, and NCBI *r *= 0.89).

The quality of genome annotations profoundly influences orthology inference. Our findings underscore that OMArk’s percentage of Unknown and Taxonomically Consistent genes are predictive of the reliability of orthology assessments. Higher-quality annotations, as indicated by low percentages of Unknown and high percentage of Taxonomically consistent genes, tend to yield more reliable orthology assignments in our results. Moreover, when comparing higher-quality annotations, subtle differences in percentage of missing genes in both BUSCO and OMArk also correlate highly with the orthology recall.

## 4. Discussion

*In silico* genome structural annotation is still a bottleneck at a time characterized by big data in genomics analyses. We assessed the orthology assignments resulting from four structural gene annotation sources: the NCBI eukaryotic genome annotation pipeline, the Ensembl gene annotation system, UniProt’s Reference Proteomes, and Augustus *ab initio*. We show differences not only between our baseline *ab initio* approach and methods that use all three types of evidence (*ab initio*, homology, and transcriptomic data) but also between established annotation resources (NCBI, UniProt, and Ensembl). The outcomes, as indicated by various quality indicators, consistently highlight distinctions among the four methods.

The number of protein-coding genes for each species is reasonably well-established for all the species used in this analysis (Tiessen *et al.* 2012, Guigó, 2023). This number therefore served as an initial evaluation of the annotations. For *ab initio*, the number of genes predicted was substantially higher than the other methods and more variable between species, while the differences and variability in UniProt, NCBI, and Ensembl were minor. Despite the small difference in the number of protein-coding genes between NCBI and Ensembl, the similarity between their gene models is only high for *H. sapiens* and *M. musculus.* Nevertheless, NCBI and Ensembl gene models are still significantly more similar than NCBI and *ab initio* models or Ensembl and *ab initio* models.

On the other hand, there is no ground truth for assessing the orthology inference results. Therefore, this assessment necessitates various metrics, such as the proportion of genes with an ortholog, the number and size of orthologous groups, and the ability of orthologs to recapitulate species trees. Our findings consistently demonstrate that our baseline annotation method (Augustus) results in poorer quality orthology inference. Even though the exact impact of using completely *ab initio* gene models in orthology inference had not been previously evaluated at the start of this study, the underperformance of Augustus in comparison with the other methods was expected. Augustus is an *ab initio* gene prediction tool, which uses a generalized hidden Markov model (HMM) for gene prediction. Extrinsic evidence such as transcriptome sequences or annotations of closely related genomes can be provided as additional files, which modify the probabilities during prediction (Stanke and Waack 2003, Hoff and Stanke 2019). However, our use of the software was inherently suboptimal, as we relied solely on Augustus’ pre-computed model parameters and only provided the soft-masked genome assembly as evidence. On the contrary, NCBI’s Eukaryotic Genome Annotation Pipeline (EGAP) and Ensembl’s gene annotation system are full genome annotation pipelines. They integrate *ab initio* gene predictions but rely less on them and integrate other evidence types, as well as manual curation in some cases [HAVANA in Ensembl and curation in RefSeq (Aken *et al.* 2016, Goldfarb *et al.* 2025)]. The poor quality orthology inference results that we obtained for Augustus here are not a direct assessment of the gene prediction method itself, but rather a finding showcasing the impact of relying on solely *ab initio* gene models for orthology prediction. Comprehensive assessment and improvement of gene models from *ab initio* prediction methods is an active area of research (Scalzitti *et al.* 2020, Holst *et al.* 2023, Vuruputoor *et al.* 2023, Abbas *et al.* 2024, Brůna *et al.* 2024, Gabriel *et al.* 2024a), and we primarily focus on the impact on orthology results. There are some popular and more recent *ab initio* prediction tools which integrate an optimized application of Augustus in their workflow, such as BRAKER2 (Brůna *et al.* 2021), BRAKER3 (Gabriel *et al.* 2024a), or GALBA (Brůna *et al.* 2023), and some which integrate machine learning and HMMs, achieving outstanding results—namely Helixer (Holst *et al.* 2023) and Tiberius (Gabriel *et al.* 2024b).

More surprisingly, there was also a significant impact of using different high-quality annotations from established sources on the quality of orthology assignments, with NCBI yielding higher quality across all evaluated metrics. The main difference between NCBI’s Eukaryotic Genome Annotation Pipeline (EGAP) and Ensembl’s Gene annotation system is the way in which they incorporate several lines of evidence into the end gene model results. While both incorporate a plethora of evidence types such as protein sequences, cDNAs and ESTs, these are used differently (Aken *et al.* 2016, Goldfarb *et al.* 2025). In NCBI’s EGAP, the alignments from all evidence types are incorporated into their gene prediction tool “Gnomon”, which is supplemented with an *ab initio* HMM-based algorithm. Finally, there is a best model selection step which again includes models coming from other sources, such as known and curated RefSeq transcripts (Goldfarb *et al.* 2025). On the other hand, the Ensembl annotation system predicts genes independently for each of its “stages” (targeted, similarity, species-specific cDNA and EST, RNA-seq reads) using the gene prediction tool “GeneWise” (Birney *et al.* 2004), in some cases in addition to previously predicted models using “Genscan”. Finally, it finds a consensus set of gene models coming from all stages. It is possible that EGAP’s approach finds more supported gene models while Ensembl’s returns less informed gene models due to its unification step allowing for gene models supported with less evidence (e.g. only one layer in the Layer Annotation step). However, this could also be a benefit, as Ensembl’s annotation system approach might be more permissive toward gene models which are only supported by one of the evidence types, such as fast-evolving genes not found using traditional homology approaches (discussed below). However, the main factor behind the difference in our results is not trivial to investigate, particularly since these pipelines include manual curation steps and the automated part is, to our knowledge, not publicly available. There is only a partial and unofficial release available for the Eukaryotic Genome Annotation Pipeline (EGAPx—https://github.com/ncbi/egapx). Moreover, the EGAP incorporates a quality assessment step which includes the assessment of the predicted number of orthologs, which might be directly related to our results. There are other popular and more recent genome annotation pipelines which are publicly available such as StringTie (RNA-seq based) (Pertea *et al.* 2015) and GeMoMa (homology based) (Keilwagen *et al.* 2019).

The metrics we used do not serve as unequivocal indicators of annotation quality. For example, the absence of orthologous proteins in other species is not necessarily indicative of erroneous protein-coding gene models. Given that new genes tend to be shorter, evolve faster, and have lower expression than well-established genes (Toll-Riera *et al.* 2009, Zhao *et al.* 2024), some of the surplus short genes inferred in *ab initio* could potentially be true lineage-specific genes. Indeed, the Ensembl and NCBI eukaryotic gene annotation pipelines have stringent requirements to include a solely *ab initio* model in their final gene set (Aken *et al.* 2016, Goldfarb *et al.* 2025). It appears that most of the *ab initio* potential lineage-specific genes are artifacts, as the number of singletons is too high considering estimates of the rate of emergence of new genes (Casola 2018, Zhang *et al.* 2019, Zile *et al.* 2020, Vakirlis *et al.* 2024). The numbers of singleton genes predicted using the NCBI, Ensembl, and UniProt gene sets fit these estimations better than the numbers using *ab initio*.

Similarly, an annotation method that yields a proteomes set which leads to less comprehensive and complete HOGs than other methods does not necessarily indicate the gene set is more “wrong”. However, the difference between the *ab initio* baseline and more “highly-trusted” annotations indicates that most protein-coding genes do have orthology and more complete and comprehensive HOGs.

The exact annotation factors that impact orthology analysis are not fully clear, yet our findings show a correlation with predicted protein length. Protein length, a direct result from gene annotation, likely influences orthology inference (Weisman *et al.* 2022). Most orthology inference methods rely on sequence alignments and often incorporate a specific alignment overlap criteria as part of the orthology algorithm [e.g. OMA (Train *et al.* 2017), OrthoDB (Kriventseva *et al.* 2019), PANTHER (Thomas *et al.* 2022)]. We find that while the proteins’ length distribution is variable between methods, the protein lengths of genes with orthologs are highly consistent across methods. The discrepancy in length primarily affects singleton genes, which are markedly shorter across all methods, but longest for NCBI. Notably, species median gene lengths correlate closely with their proportion of orthologs, with overall shorter sequences tending to have fewer orthologs. This pattern indicates that departures from the typical eukaryotic protein length distributions, indicative of low proteome or genome quality (Nevers *et al.* 2023), indeed pose challenges for orthology inference. Consequently, protein length, a product of gene annotation, directly influences orthology results.

Many short proteins are often a sign of assembly or annotation fragmentation, which hampers correct orthology inference either by not matching a fragment to a longer protein or by inferring separate, incomplete orthologous groups comprised of gene fragments. Such fragmentation not only affects the accuracy of orthology assignments but can also introduce erroneous domain annotations, which can result in incorrect conclusions about domain gain and loss events. In non-human primates, annotation errors led to the detection of up to 10 times the actual number of domain loss events and up to three times the actual number of domain gain events (Kress *et al.* 2023). It is likely that the extra number of genes in some proteomes of our analysis, aside from spurious annotations, are caused by fragmentation.

Another factor that influences orthology is the interaction of the annotation source with different orthology inference software. We accounted for this by repeating the Generalized Species Tree Discordance Benchmark using orthology assignments obtained with OrthoFinder (Emms and Kelly 2019). Although the results generally agree, there was a swap in the relative positions of UniProt and Ensembl in terms of recall. This was especially surprising as most of the UniProt proteomes in our analysis were derived from Ensembl. One major difference between Ensembl and UniProt proteomes is that UniProt selects only one of the isoforms provided by Ensembl as their canonical isoform, and one major difference between OrthoFinder and OMA is their isoform handling approach. OrthoFinder selects the longest isoforms, and OMA opts for the “evolutionarily best conserved isoforms” (Altenhoff *et al.* 2021). We found that the difference between OMA’s and OrthoFinder’s UniProt and Ensembl results were largely due to their isoform selection. Isoform choice influences sequence comparisons, the delineation of orthologs and paralogs, and gene tree construction. The fact that such differences could be detected further reinforces the importance of carefully selecting annotation and orthology inference software for evolutionary analyses.

This study sought to replicate the initial stages of a typical comparative genomics investigation, examining the outcomes of orthology inference using different well-established annotation sources. Although we used four well-known sources, countless combinations of methods and parameters exist for gene annotation pipelines. The 20 Chordata genomes included here are among the most studied across the eukaryotic domain. However, some NCBI and Ensembl annotations used were not the latest versions due to the need for consistency in the assemblies. Therefore, our results might not be representative of the overall annotation performance of these resources. Nevertheless, we are likely using some of the most accurate annotations across metazoan genomes. Our findings indicate that the choice of annotation method affects orthology results, with noticeable performance differences even among high-quality sources. This suggests that less-studied or lower-quality genomes annotated by diverse pipelines could render even more discordant orthology inference results. The *ab initio* method, Augustus, which showed the most significant differences, served as a baseline annotation method. Although this method generally performed poorly, the choice of a close well annotated species’ parameters was crucial.

Downstream analyses such as comparative, functional, and evolutionary genomics are highly impacted by the quality of orthology assignments (Altenhoff *et al.* 2016), which we find is in turn impacted by the gene annotation method. Poor orthology results can lead to erroneous conclusions, such as false gene losses and lineage-specific inferences (Weisman *et al.* 2022). We observed that some methods tend to predict more genes which ultimately would not be related by orthology—due to substandard gene models caused by fragmentation or erroneous annotations. By relying on these orthology inferences, comparative genomics and evolutionary biology studies could result in artifactual findings, potentially affecting phylogenetic studies, drug discovery, and disease research that use model organisms, and gene function propagation.

Our findings call for awareness on the impact of annotation on orthology inference and downstream analyses. The complexity of structural genome annotation is well recognized, and there are emerging efforts within the scientific community to evaluate and standardize annotation methods, such as those of the ERGA Annotation Committee (Brown *et al.* 2024). Yet, community-wide standards for genome annotation are still not fully established. We recommend integrating BUSCO and OMArk (Manni *et al.* 2021, Nevers *et al.* 2024) into annotation workflows or when selecting annotations. As a first step, we suggest focusing on the percentage of Unknown genes and Taxonomically consistent genes, whereas gene completeness and missingness can guide the choice between higher-quality annotations. Moreover, we propose evaluating annotations through orthology-based benchmarks such as the proportion of genes with orthologs, the Generalized Species Tree Discordance Benchmark, or gene families (HOGs) quality assessments.

Nevertheless, homology inference has its own limitations, such as high domain complexity, fast-evolving genes, and short proteins. Homology inference methods usually struggle to align genes with naturally low alignment scores due to their biological properties, such as fast-evolving genes like immunity genes or hybrid sterility genes (Trachana *et al.* 2011, Schwartz *et al.* 2014, Vinkler *et al.* 2023). Genes with complex domain evolution, such as mucins, and short proteins (approximately shorter than 200 aa long), also hamper correct automatic alignments and often result in erroneous orthology assignments (Trachana *et al.* 2011). Therefore, homology-based annotation evaluation approaches, such as the metrics we propose, BUSCO, and OMArk, should be used together with other approaches, such as protein length distribution comparisons (Nevers *et al.* 2023), gene model summary statistics (Brown *et al.* 2024), mapping to a reference annotation (Pertea and Pertea 2020), ratio of mono-exonic to multi-exonic genes (Vuruputoor *et al.* 2023), or novel machine learning approaches such as Psauron (Sommer *et al.* 2025). Failing to do so might favor genes annotated using homology pipelines at the expense of true genes that can only be found using either transcriptomic or *ab initio* evidence.

Compared to existing homology-based annotation quality assessment methods, the orthology-based metrics we propose here have the advantage of being reference-free, therefore overcoming the problem of reference bias. Here, we provide orthology assignment metrics for comparison, and plan to integrate them as part of the Quest for Orthologs Benchmarking web service (Altenhoff *et al.* 2016).

## Supplementary Material

btaf365_Supplementary_Data

## Data Availability

All scripts and jupyter notebooks used to download the assemblies and annotations, and to run GffCompare, Augustus, OMA, OrthoFinder, benchmarks, and additional analyses can be found on GitHub (https://github.com/DessimozLab/Annotation-Orthology). Complete annotation sets and the orthology results can be found at Zenodo (https://doi.org/10.5281/zenodo.10907108).
