# Supplementary material for: Annotation matters: the effect of structural gene annotation on orthology inference
Source: Bioinformatics. 2025 Jun 25;41(7):btaf365. doi: 10.1093/bioinformatics/btaf365 (PMC12263111; doi:10.1093/bioinformatics/btaf365)
Supplement: btaf365_Supplementary_Data [file btaf365_supplementary_data.zip › Supplementary_Material_AnnotationMatters_Bioinformatics.pdf]

Supplementary Material for “Annotation matters: the effect of structural gene annotation on orthology”

General information about assemblies and annotations ………………………… 2

Number of genes ……………………………………………………………… 3
Supplementary Results

Comparison of whole genome protein-coding gene annotations with GffCompare … 4
Supplementary Results

Proportion of orthologous genes ……………………………………………………… 6
Supplementary Results

Singletons and estimated number of lineage-specific genes ………………………… 7
Supplementary Methods
Supplementary Results

Assessment of Hierarchical Orthologous Groups (HOGs) …………………………. 9
Supplementary Results

Generalized Species Tree Discordance Benchmark …………………………………. 10
1. Supplementary Methods
Primary and top-level assemblies
OrthoFinder
Isoform Handling
2. Supplementary Results

Gene length ………………………………………………………………………. 14
Supplementary Results

BUSCO and OMArk Results ………………………………………………………. 19

Supplementary References ……………………………………………………………. 27

General information about assemblies and annotations

Table S1. Species included in the analyses, information about their assembly and annotation accessions, and the species parameters used in Augustus for each species (*).

Species name	Common name	NCBI Taxon ID	Assembly name	Assembly ID	NCBI Refseq (Annotation)	Uniprot RefProt ID	Ensembl assembly & annotation release	Augustus parameters species
Ciona intestinalis	Vase tunicate	7719	KH	GCA_000224145.1	GCF_000224145.1	UP000008144	108.3	ciona
Erpetoichthys calabaricus	Reedfish	27687	fErpCal1.1	GCA_900747795.2	GCF_900747795.1	UP000694620	108.11	zebrafish
Lepisosteus oculatus	Spotted gar	7918	LepOcu1	GCA_000242695.1	GCF_000242695.1	UP000018468	108.1	zebrafish
Danio rerio	Zebrafish	7955	GRCz11	GCA_000002035.4	GCF_000002035.6	UP000000437	106.11	zebrafish
Salmo trutta	Brown trout	8032	fSalTru1.1	GCA_901001165.1	GCF_901001165.1	UP000472277	108.11	zebrafish
Gadus morhua	Atlantic cod	8049	gadMor3.0	GCA_902167405.1	GCF_902167405.1	UP000694546	108.3	zebrafish
Echeneis naucrates	Live sharksucker	173247	fEcheNa1.1	GCA_900963305.1	GCF_900963305.1	UP000472264	108.1	zebrafish (*)
Oryzias latipes	Japanese rice fish/ medaka fish	8090	ASM223467v1	GCA_002234675.1	GCF_002234675.1	UP000001038	108.1	zebrafish (*)
Xenopus tropicalis	Western clawed frog	8364	UCB_Xtro_10.0	GCA_000004195.4	GCF_000004195.4	UP000008143	108.10	human
Podarcis muralis	Wall lizard	64176	PodMur_1.0	GCA_004329235.1	GCF_004329235.1	UP000472272	108.1	chicken
Chrysemys picta bellii	Painted turtle	8478	Chrysemys_picta_bellii-3.0.3	GCA_000241765.2	GCF_000241765.3	UP000694380	108.303	chicken
Gallus gallus	Chicken	9031	GRCg6a	GCA_000002315.5	GCF_000002315.6	UP000000539	102.6	chicken
Taeniopygia guttata	Zebra finch	59729	bTaeGut1_v1.p	GCA_003957565.2	GCF_003957565.1	UP000007754	108.12	chicken
Strigops habroptila	Kakapo	2489341	bStrHab1_v1.p	GCA_004027225.1	GCF_004027225.1	UP000472266	108.11	chicken
Ornithorhynchus anatinus	Platypus	9258	mOrnAna1.p.v1	GCA_004115215.2	GCF_004115215.1	UP000002279	108.1	human
Sarcophilus harrisii	Tasmanian devil	9305	mSarHar1.11	GCA_902635505.1	GCF_902635505.1	UP000007648	108.1	human
Phocoena sinus	Vaquita	42100	mPhoSin1.pri	GCA_008692025.1	GCF_008692025.1	UP000694554	108.1	human
Felis catus	Domestic cat	9685	Felis_catus_9.0	GCA_000181335.4	GCF_000181335.3	UP000011712	108.9	human
Mus musculus	Mouse	10090	GRCm38.p6	GCA_000001635.8	GCF_000001635.26	UP000000589	102.38	human
Homo sapiens	Human	9606	GRCh38.p12	GCA_000001405.27	GCF_000001405.38	UP000005640	97.38	human

(*) For *Oryzias latipes* and *Echeneis naucrates,* the evolutionarily closest species available in Augustus was *Xiphophorus maculatus.* However, when selecting this species, the total number of genes predicted were only 39 and 29, respectively, so we ultimately opted to use zebrafish (*D. rerio*) parameters for these two species.

Number of genes

Among the four main methods, *ab initio* (Augustus) predicted the most variable number of genes for different species (Table S2), likely due to the species for which the parameters had been trained on. Notably, Augustus predicted fewer genes than the other methods for three species– *D. rerio, C. intestinalis,* and *S. harrisii (*Tasmanian devil*)*- two of which are species Augustus was trained on (*D. rerio* and *C. intestinalis*). Conversely, two species had an extremely high number of proteins in the *ab initio* predictions, *E. calabaricus* (reedfish) and *C. picta bellii* (western painted turtle) (Fig. 1a). This discrepancy between *ab initio* and the other methods was also notable for *X. tropicalis* (western clawed frog) and *P. muralis* (common wall lizard). In Aves and Mammalia, the number of predicted proteins is relatively stable among all methods, likely because a bird and mammal (*Gallus gallus* and *Homo sapiens*) were used for training Augustus. Indeed, there is a correlation between the divergence time of the species being annotated relative to the species used for training and the number of inferred proteins (Pearson's r=0.68 , p=0.001; Fig. S1).

Table S2. Summary statistics for number of protein-coding genes and proportion of proteins with inferred orthology across 20 genomes.

Method	Median Protein number	Mean Protein number	Standard Deviation	Median Orthology Proportion	Mean proportion of proteins with orthology	Standard Deviatio n
Ab initio	23451	32269	20717	0.70	0.65	0.17
Ensembl	21420	21836	6218	0.93	0.91	0.11
NCBI	20699	21342	5840	0.94	0.92	0.08
UniProt	20881	21410	5769	0.91	0.90	0.11

Table S3. Wilcoxon signed-rank test of number of genes between methods. A small p-value in a Wilcoxon signed-rank test means you have enough evidence to reject the null hypothesis in favor of the alternative.

Method 1	Method 2	p-value	Statistic	Alternative Hypothesis
Ab initio	UniProt	0.0014	182	"greater"
Ab initio	Ensembl	0.0021	179	"greater"
Ab initio	NCBI	0.0007	186	"greater"
UniProt	Ensembl	0.0018	30	"less"
UniProt	NCBI	0.3781	114	"greater"
Ensembl	NCBI	0.0825	143	"greater"

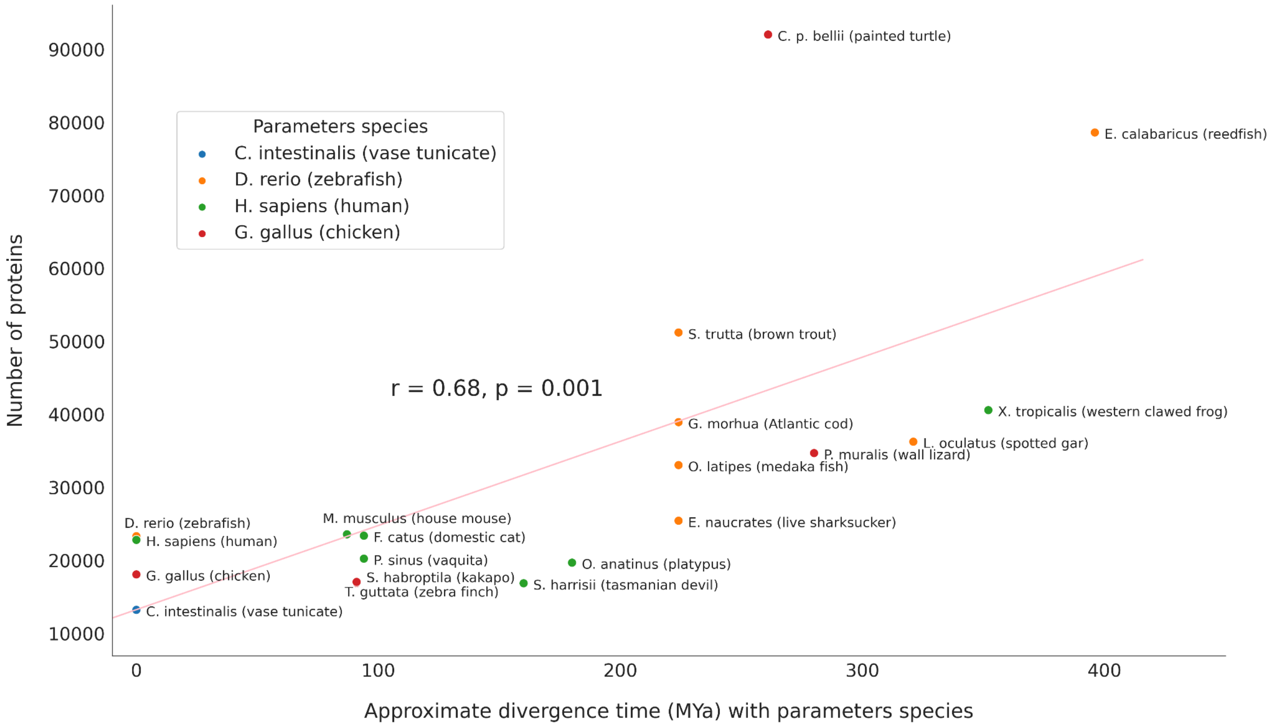

Figure S1. Relationship between Augustus number of proteins and estimated divergence time with species used for training the parameters.

Comparison of whole genome protein-coding gene annotations with GffCompare

Table S4. Wilcoxon signed-rank test of Jaccard indexes between method pairs.

Method pair 1	Method pair 2	p-value	Statistic	Alternative Hypothesis
Ab initio - Ensembl	*Ab initio* – NCBI	0.31	91	“less”
Ab initio - Ensembl	Ensembl - NCBI	9.54e-07	0	“less”
Ab initio - NCBI	Ensembl - NCBI	9.54e-07	0	“less”

Table S5. Table with Jaccard indexes of both computations for each method pair. Jaccard index 1 corresponds to the computation with the first method as reference, and Jaccard index 2 with the second method as reference.

Species	Method Pair	Jaccard index 1	Jaccard index 2	Species	Method Pair	Jaccard index 1	Jaccard index 2
Chrysemys picta bellii	Augustus-Ensembl	0.0182	0.0178	Mus musculus	Augustus-Ensembl	0.0680	0.0652
	Augustus-NCBI	0.0227	0.0223		Augustus-NCBI	0.0650	0.0620
	Ensembl-NCBI	0.2764	0.2758		Ensembl-NCBI	0.8788	0.8801
Ciona intestinalis	Augustus-Ensembl	0.0685	0.0685	Ornitho-rhynchus anatinus	Augustus-Ensembl	0.0552	0.0552
	Augustus-NCBI	0.1203	0.1132		Augustus-NCBI	0.0540	0.0530
	Ensembl-NCBI	0.1846	0.1796		Ensembl-NCBI	0.3022	0.2991
Danio rerio	Augustus-Ensembl	0.0985	0.0971	Oryzias latipes	Augustus-Ensembl	0.0580	0.0580
	Augustus-NCBI	0.0866	0.0851		Augustus-NCBI	0.0564	0.0552
	Ensembl-NCBI	0.5540	0.5525		Ensembl-NCBI	0.3572	0.3567
Echeneis naucrates	Augustus-Ensembl	0.0629	0.0629	Phocoena sinus	Augustus-Ensembl	0.0536	0.0532
	Augustus-NCBI	0.0772	0.0762		Augustus-NCBI	0.0506	0.0484
	Ensembl-NCBI	0.3191	0.3175		Ensembl-NCBI	0.4342	0.4305
Erpetoichthys calabaricus	Augustus-Ensembl	0.0100	0.0098	Podarcis muralis	Augustus-Ensembl	0.0465	0.0465
	Augustus-NCBI	0.0103	0.0099		Augustus-NCBI	0.0466	0.0455
	Ensembl-NCBI	0.3144	0.3124		Ensembl-NCBI	0.2022	0.1997
Felis catus	Augustus-Ensembl	0.0629	0.0623	Salmo trutta	Augustus-Ensembl	0.0594	0.0596
	Augustus-NCBI	0.0508	0.0489		Augustus-NCBI	0.0585	0.0576
	Ensembl-NCBI	0.3531	0.3471		Ensembl-NCBI	0.3436	0.3393
Gadus morhua	Augustus-Ensembl	0.0462	0.0464	Sarcophilus harrisii	Augustus-Ensembl	0.0419	0.0412
	Augustus-NCBI	0.0459	0.0452		Augustus-NCBI	0.0589	0.0562
	Ensembl-NCBI	0.2476	0.2446		Ensembl-NCBI	0.3545	0.3534
Gallus gallus	Augustus-Ensembl	0.0749	0.0741	Strigops habroptila	Augustus-Ensembl	0.0563	0.0559
	Augustus-NCBI	0.0808	0.0800		Augustus-NCBI	0.0790	0.0778
	Ensembl-NCBI	0.4089	0.4085		Ensembl-NCBI	0.3282	0.3272
Homo sapiens	Augustus-Ensembl	0.0697	0.0674	Taeniopygia guttata	Augustus-Ensembl	0.0538	0.0539
	Augustus-NCBI	0.0659	0.0639		Augustus-NCBI	0.0752	0.0734
	Ensembl-NCBI	0.8950	0.8958		Ensembl-NCBI	0.2826	0.2788
Lepisosteus oculatus	Augustus-Ensembl	0.0307	0.0309	Xenopus tropicalis	Augustus-Ensembl	0.0175	0.0160
	Augustus-NCBI	0.0231	0.0228		Augustus-NCBI	0.0187	0.0179
	Ensembl-NCBI	0.2508	0.2499		Ensembl-NCBI	0.3886	0.3798

Proportion of orthologous genes

Table S6. Wilcoxon signed-rank test results comparing proportion of orthologous genes between methods.

Method 1	Method 2	p-value	Statistic	Alternative Hypothesis
Ab-initio	UniProt	2.86e-06	2	"less"
Ab-initio	Ensembl	1.91e-06	1	"less"
Ab-initio	NCBI	9.54e-07	0	"less"
UniProt	Ensembl	6.67e-05	12	"less"
UniProt	NCBI	6.67e-05	12	"less"
Ensembl	NCBI	0.0068	40	"less"

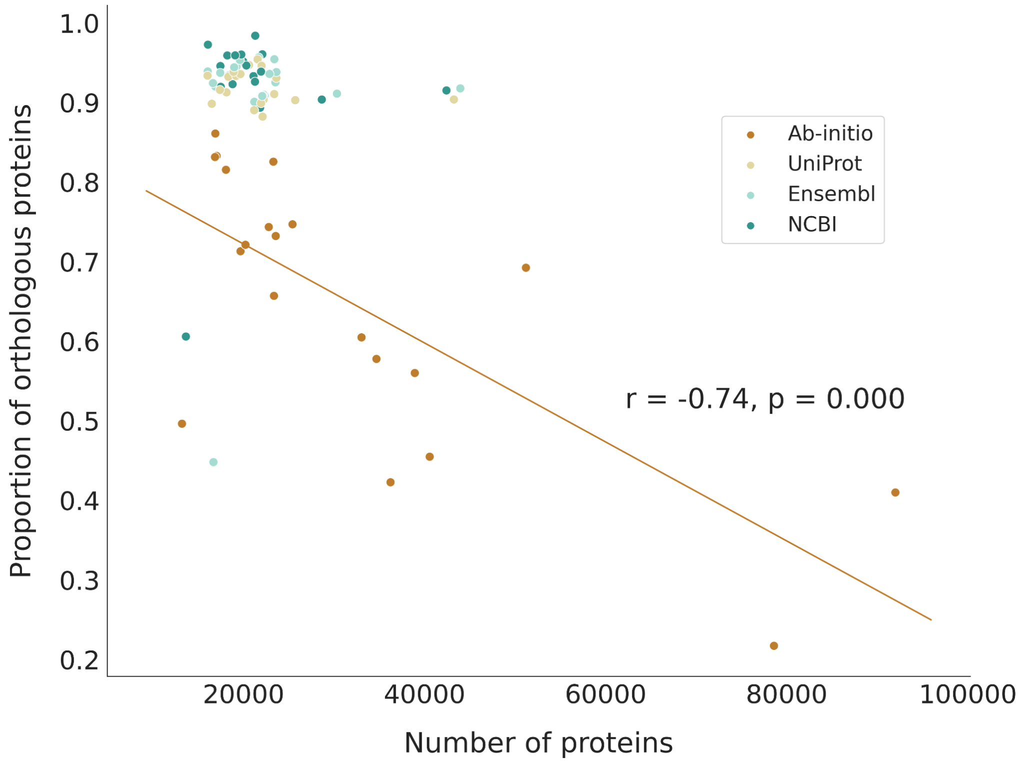

Figure S2. Relationship between the number of genes and the proportion of orthologs for each species across annotation methods. The correlation values correspond to the Pearson's correlation between Augustus *ab initio*'s gene numbers and their corresponding proportion of genes with orthology.

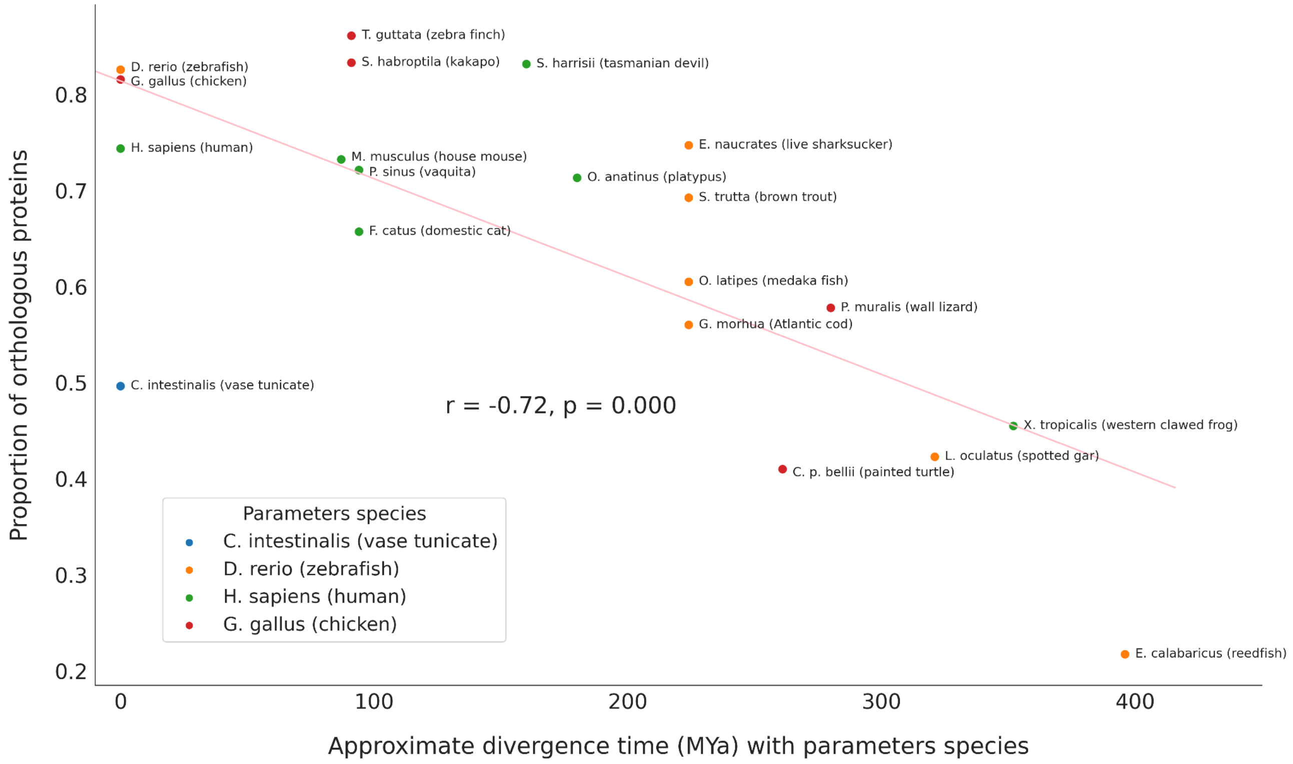

Figure S3. Relationship between Augustus proportion of orthologous proteins and divergence time with species used for training the parameters.

Singletons and estimated number of lineage-specific genes

Supplementary Methods

We estimated the expected number of taxonomically restricted (i.e. lineage-specific) genes for each species by multiplying its divergence time from the phylogenetically closest species in the dataset and published de novo gene emergence rates. Because these rates vary among clades, we bracketed our expectations using an upper bound of 51.5 new genes per million years (MY) based on (Zhang et al. 2019), and a lower bound of 7.4/MY based on (Vakirlis et al. 2024), as they found 37 novel genes in *S. cerevisae*, which diverged 4.98 MYA from its closest relative *S. paradoxus.* Other studies point towards the lower end of this range (Casola 2018; Zile et al. 2020). The estimated divergence times were obtained from the TimeTree 5 project (Kumar et al. 2022).

Supplementary Results

For all species in NCBI, Ensembl and UniProt, the number of singletons (genes without orthologs) falls either within the lower end of the ranges in number of expected de novo genes or below it. However, for *ab initio,* 13/20 genomes show a higher number than the upper bound of the range (Fig. S4, Supplementary Table 11). Of the remaining 7 *ab initio* genomes whose singleton counts did fall within the expected range, only *C. intestinalis* (600 MY divergence) has a lower number of genes without orthology than another method (UniProt).

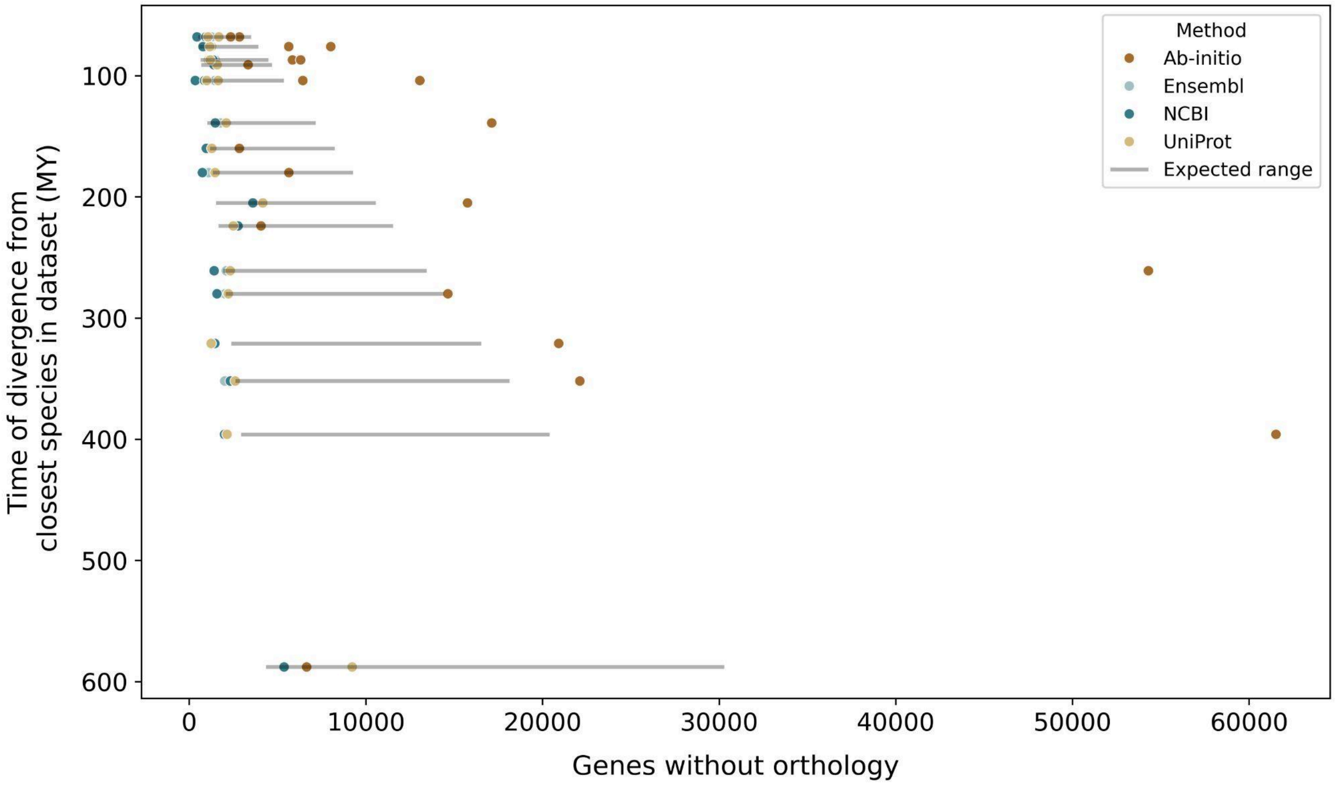

Figure S4. Genes without orthology compared to the expected number of taxonomically restricted genes based on estimated de novo gene emergence rates. The upper bound corresponds to a de novo gene emergence rate of 51.5 new genes per MY, while the lower bound is based on a rate of 7.4 new genes/MY.

Assessment of Hierarchical Orthologous Groups (HOGs)

Table S7. RootHOGs size summary statistics: total number (n), median and average sizes with the corresponding standard deviation, number of rootHOGs with 20 single copy genes and number of rootHOGs with 2 genes.

Method	n	Median size	Avg. size	St. Dev	n of single-copy 20-sized HOGs	n of 2-sized HOGs
Ab-initio	45,153	4	7.674	19.338	19	15,095
UniProt	30,658	8	12.281	18.713	283	6,334
Ensembl	30,576	8	12.711	18.300	250	6,167
NCBI	26,678	11	14.522	47.657	621	4,556

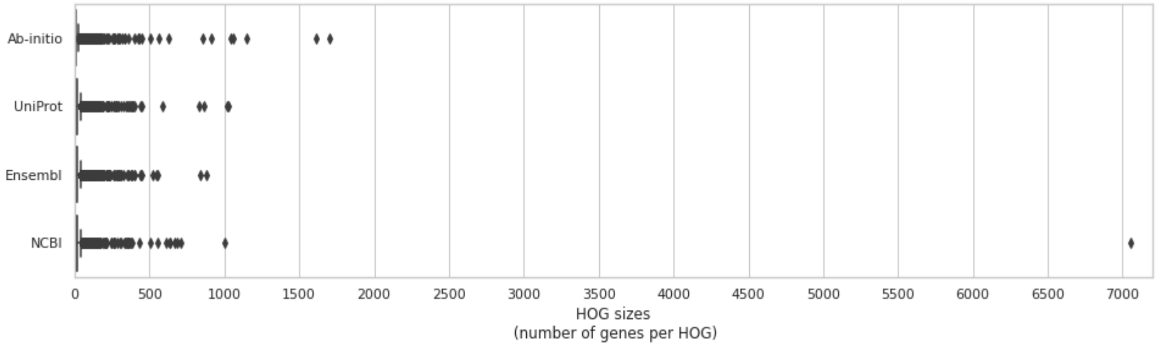

Figure S5. RootHOG size distribution (number of genes per HOG). Version of Figure 4b showing outliers.

Generalized Species Tree Discordance Benchmark

1. Supplementary Methods

Primary and top-level assemblies (sequences)

Three species (*Homo sapiens*, *Mus musculus* and *Danio rerio*) have two different assembly types: the "top-level" and the "primary" assembly. The primary assembly consists of the reference sequences of the genome, while the top-level assembly includes these as well as haplotypes and patches, also called alternative sequences.

The NCBI and Ensembl annotations derive from top-level assemblies. However, due to the redundancy of these assemblies and the unnecessary computational time and resources that entails annotating them, we ran Augustus over the primary assemblies of these species. Therefore, to ensure an appropriate comparison and the independence of our results from the assembly type, we derived two extra proteome sets for Ensembl and NCBI. To do so, we filtered the NCBI and Ensembl proteome sets to include only genes which are present only in the primary assembly sequences. In the main text, we use the "top-level" results, as it is the default proteome set obtained from NCBI and Ensembl annotations.

We additionally ran and evaluated the orthology inference for the primary assembly proteomes in the same way as for the other proteome sets, running OMA independently twice (once for Ensembl primary assembly and once for NCBI primary assembly). We subsequently ran the Generalized Species Tree Discordance Benchmark on these two additional sets.

OrthoFinder Methods

OrthoFinder v2.5.4 (Emms and Kelly 2019) was executed once for each annotation method's proteomes, using default parameters. Therefore, we ran it six times, four for the standard annotation methods sets, and twice for the primary assembly sets described above. For genes with alternative splicing isoforms, OrthoFinder handles only one. Therefore, we identified the longest isoforms for these cases using the script "primary_transcript.py" provided by OrthoFinder, or a slightly modified version for the NCBI proteomes (primary_transcript_idcorr.py, available at the github repository).

Isoform handling Methods

Two of the annotation methods covered by our analyses (NCBI and Ensembl) provide more than one splicing variant, or alternative splicing isoforms, for many genes. OrthoFinder (and most orthology inference methods) chooses the longest isoform as the "canonical isoform" of each gene. while OMA identifies the most evolutionarily conserved isoform as the canonical one. To do so, the OMA standalone pipeline needs to be provided with ".splice" files, which specify which of the fasta sequences are isoforms corresponding to the same gene. Then OMA keeps all of the isoforms and includes them in the all-against-all alignment phase of the algorithm, in which all proteins are aligned against each other. Once this is done, each gene's isoform with the best matches across all species is chosen as the representative isoform (Altenhoff et al. 2021).

We benchmarked four additional orthology sets to assess the impact of the isoform handling approach. We obtained these two of the four additional orthology sets by running OMA using the NCBI and Ensembl gene repertoires, but only with the longest isoform of each gene. The other two additional sets correspond to OrthoFinder runs over the isoforms chosen by OMA as the most conserved ones.

Therefore, the same gene repertoires were used to infer orthology from OrthoFinder and OMA for comparison in the Generalised Species Tree Discordance benchmark.

2. Supplementary results

The OMA results were virtually identical whether using top-level or primary assemblies in recall (Fig. S5; completed gene trees for NCBI top-level: 14,277, and for NCBI primary: 14,176; completed gene trees for Ensembl top-level: 9,271, and for Ensembl primary: 9,116) as well as in accuracy (Fig. S5; mean RF for NCBI top-level: 0.216, and for NCBI primary: 0.216; mean RF for Ensembl top-level: 0.225 and for Ensembl primary: 0.226). The differences are likely due to the randomness of the benchmark when choosing genes.

The pattern in the OrthoFinder results closely resembles that of OMA. In the same way as in the OMA results, NCBI achieved the highest recall (19,719 completed gene trees) and accuracy (mean RF distance: 0.206) as illustrated in Figure S5. Secondly, *ab initio* also showed the lowest recall (10,526 gene trees) and accuracy (mean RF: 0.349). Similarly to the OMA results, UniProt's and Ensembl's recall and accuracy lie between *ab initio* and NCBI. However, in contrast to the OMA results, UniProt's recall is higher than Ensembl's in the OrthoFinder results. We found this is largely due to the isoform handling approach used, as when applying OrthoFinder's longest isoform approach in OMA, the recall for Ensembl (8,210 gene trees) highly resembles UniProt's (8,402).

In more general terms, choosing the longest isoform returns lower recall in comparison to choosing the most evolutionarily conserved isoform, both for Ensembl (8,210 and 9,271, respectively) and NCBI (12,733 and 14,277) and marginally improves their accuracy (Table S6, Fig. S5).

Regarding the primary and top-level assemblies in the OrthoFinder results, their metrics are again highly similar. The recall for NCBI top-level was 19,719 gene trees and for NCBI primary, 19,779 trees; while for Ensembl top-level it was 14,567 trees and for its primary assembly counterpart it was 14,699 trees. In terms of tree error, NCBI top-level obtained a mean RF distance of 0.206 and NCBI primary a mean of 0.210, while Ensembl top-level scored an error of 0.231 and Ensembl primary of 0.238.

Table S8. Generalized Species Tree Discordance Benchmark results for all protomes sets. The recall is the number of completed gene tree samples out of 50,000 trials, and the error is the average Robisons-Foulds distance between the gene trees and the species tree (error has been rounded up to four decimal points).

	**OMA**		**OrthoFinder**	
Annotation Method	Recall (gene trees)	Error (mean RF)	Recall (gene trees)	Error (mean RF)
**NCBI (top-level, most conserved isoforms)**	14,277	0.2157	21,736	0.2104
**NCBI primary assembly (default isoform method)**	14,176	0.2163	19,779	0.2097
**Ensembl (top-level, most conserved isoforms)**	9,271	0.2249	16,714	0.2315
**Ensembl primary assembly (default isoform method)**	9,116	0.2260	14,699	0.2379

**UniProt**	8,402	0.2205	16,954	0.2372
***Ab initio* (Augustus)**	1,462	0.2742	10,526	0.3488
**NCBI longest isoforms (top-level)**	12,733	0.2040	19,719	0.2058
**Ensembl longest isoforms (top-level)**	8,210	0.2153	14,567	0.2306

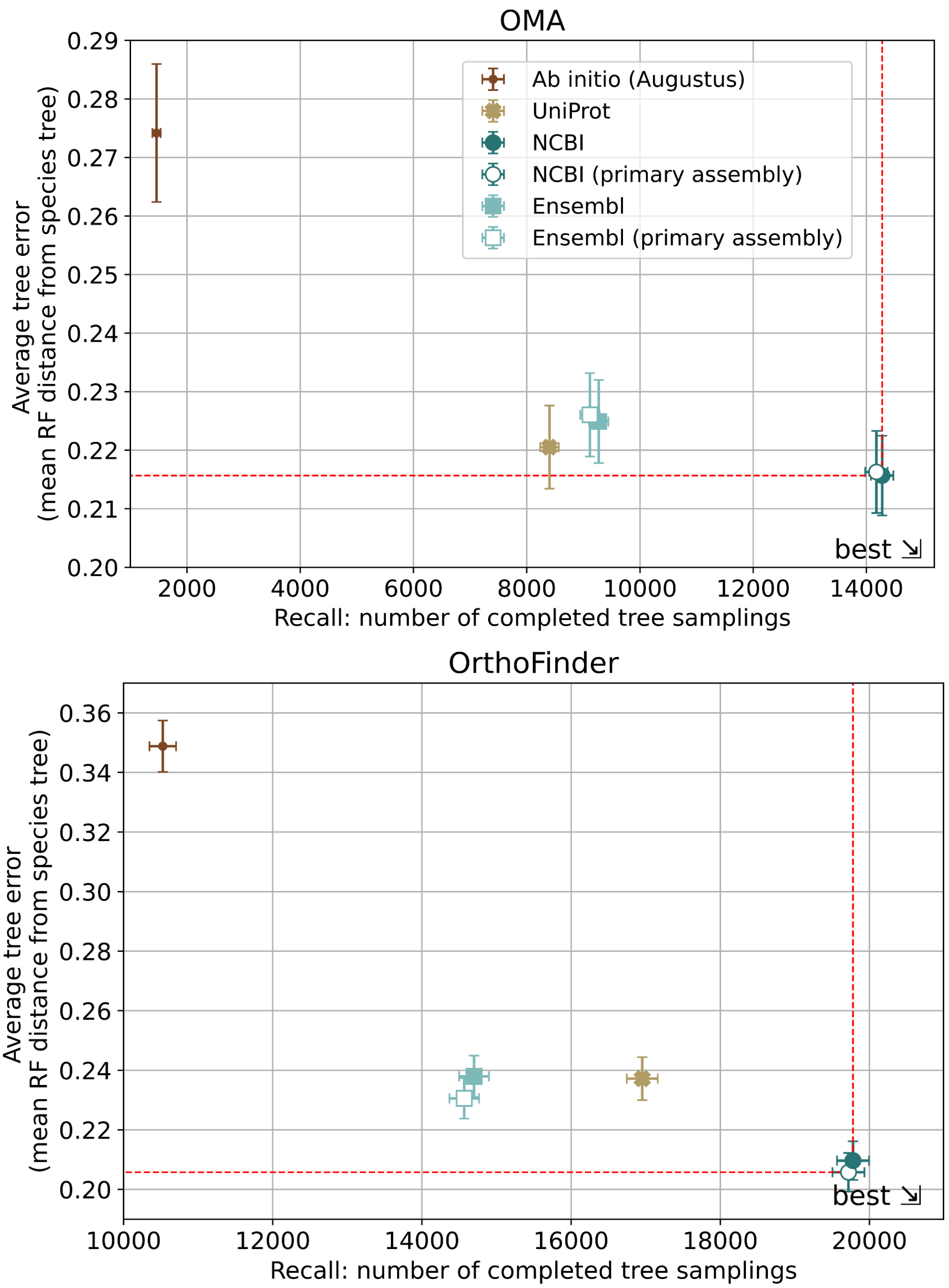

Figure S6. Species tree discordance benchmark results for all the six proteome sets, including primary assembly proteome sets, using OMA orthology (top) and OrthoFinder (bottom).

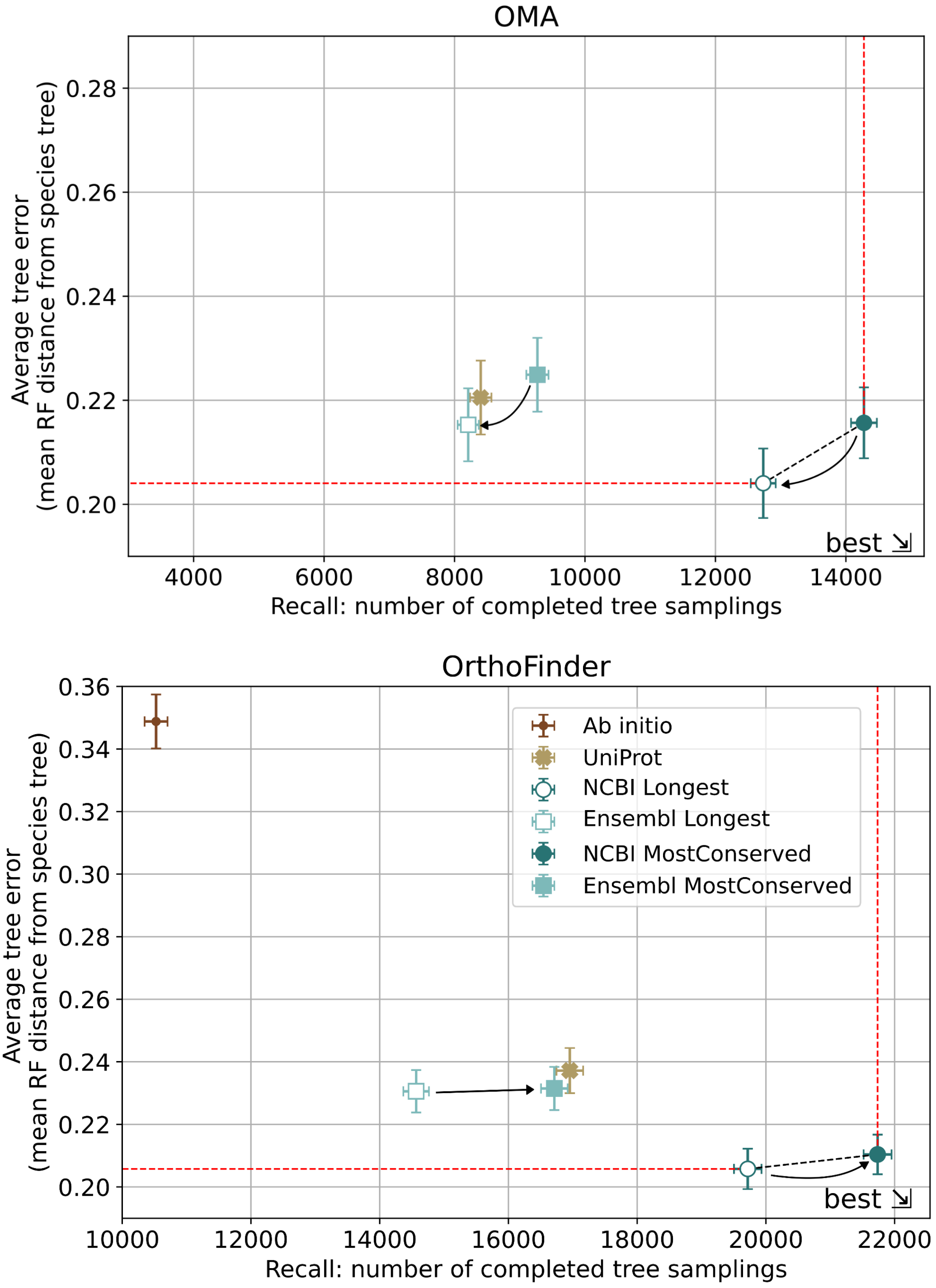

Figure S7. Species tree discordance benchmark results for the two isoform choice methods.

Gene length

Table S9. Gene length statistics

Method	count	mean	std	min	25%	50%	75%	max
Ab-initio	645,378	431.0	510.3	1	155	286	519	44,005
Ensembl	436,713	524.1	484.1	2	242	396	640	32,359
NCBI	426,843	572.3	586.8	24	270	422	685	37,665
UniProt	428,196	543.0	518.5	2	249	409	664	35,213
Primary Ensembl	428,682	525.0	484.4	2	242	397	642	32,359
Primary NCBI	424,000	571.9	586.2	24	270	422	685	37,665

Table S10. Summary statistics for genes with inferred orthology and for singletons.

Protein group	Method	count	Percentage	mean	std	min	25%	50%	75%	max
**Proteins with orthology**	Ab-initio	365’956	56.70%	571.1	588.4	54	256	409	689	37’618
	Ensembl	397’347	90.99%	550.0	491.8	50	267	419	669	32’359
	NCBI	395’108	92.57%	587.9	597.3	50	284	435	704	37’665
	UniProt	385’244	89.97%	572.1	529.4	50	278	434	695	35’213
	Primary Ensembl	389’775	90.92%	551.2	492.2	50	268	420	671	32’359
	Primary NCBI	392’836	92.65%	587.4	596.9	50	284	435	704	37’665
**Singletons**	Ab-initio	279’422	43.30%	247.6	297.8	1	111	164	273	44’005
	Ensembl	34’974	8.01%	240.1	242.2	2	100	173	292	9’786
	NCBI	25’780	6.04%	362.2	358.6	24	174	264	430	13’171
	UniProt	42’952	10.03%	281.8	301.1	2	114	198	348	7’963
	Primary Ensembl	34’721	8.10%	240.0	241.8	2	101	173	292	9’786
	Primary NCBI	25’337	5.98%	361.7	358.3	24	174	264	429	13’171

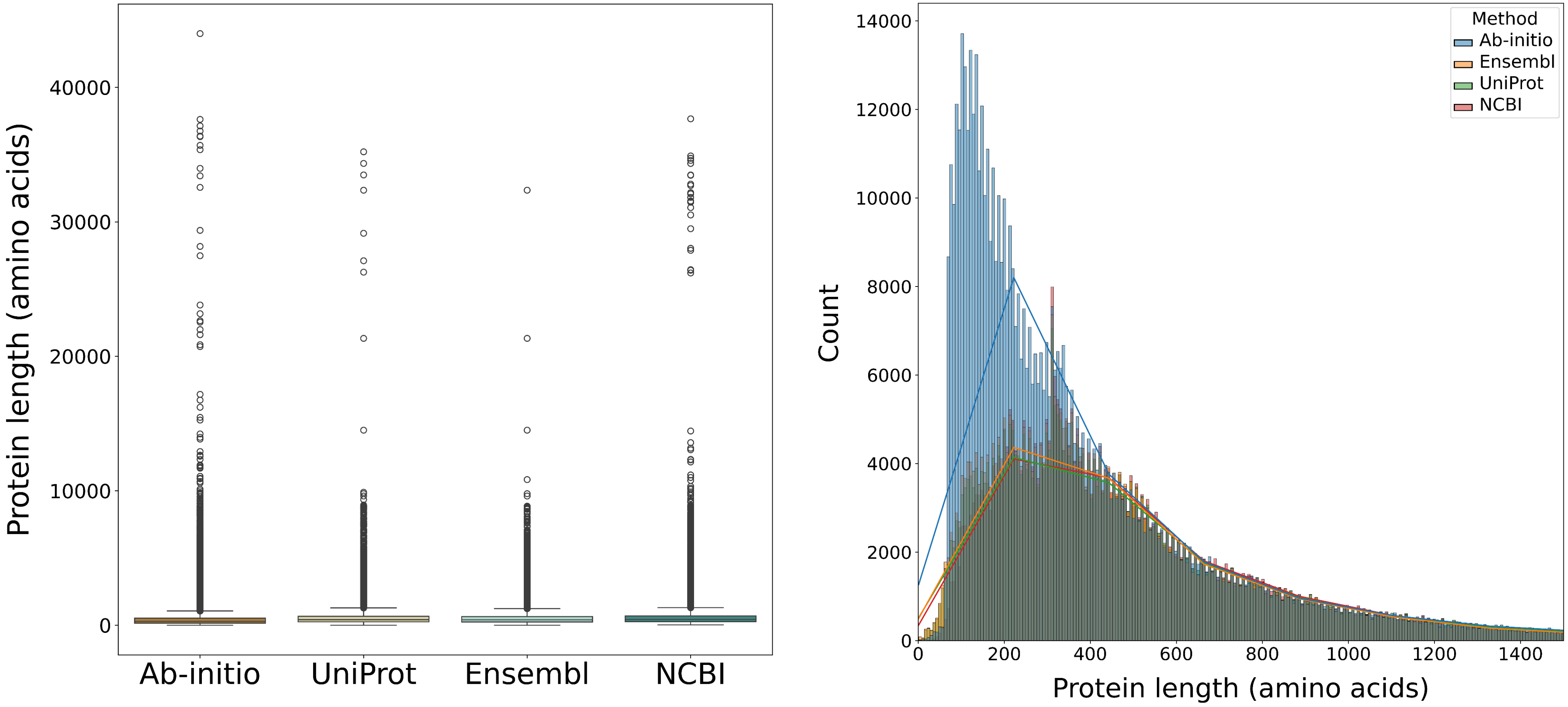

Figure S8. Protein length distribution. (Left) Protein length distribution of canonical proteins (those used in OMA) for the 4 main methods. (Right) Zoom in on "short" proteins' (<1500 amino acids) length distribution.

Protein length comparison between species and methods

NCBI displays the most consistent distributions among species and the longest proteins in general (Fig. S7, Fig. S8). There are four species for which NCBI proteins are clearly smaller than those obtained with Ensembl or UniProt: *X. tropicalis*, *G. gallus, T. guttata* and *O. anatinus* (Fig. 6a, Fig. S8). The UniProt proteomes, in these cases, contain slightly longer proteins than NCBI. Five other species exhibit higher length medians but also larger interquartile ranges in UniProt compared to NCBI, namely *P. muralis, S. harrisii, F. catus, M. musculus* and *H. sapiens*. This indicates that UniProt yields longer proteins but also more short proteins, while NCBI's proteomes show greater consistency around the median value.

Conversely, *ab-initio* shows the most significant variation between species, not only in median values but also in terms of distribution shapes (Fig. S7 and Fig. S8). *E. calabaricus* is an extreme example with the lowest median among the proteomes and a much more skewed distribution. Other examples include *L. oculatus*, *C. picta bellii*, X. tropicalis, *P. muralis, G. morhua*, *O. latipes* and to a lesser extent *F. catus.* These species' protein length rankings match those with the lower orthology percentages in section 3.2.1. Remarkably, in the case of *C. intestinalis*, *ab-initio* exhibits the longest proteins, while Ensembl and UniProt produce the shortest. NCBI, in this case, has a slightly lower median than *ab-initio* but a smaller interquartile range. The *ab-initio* zebrafish and tasmanian devil show more long proteins than the other methods but a wider interquartile range.

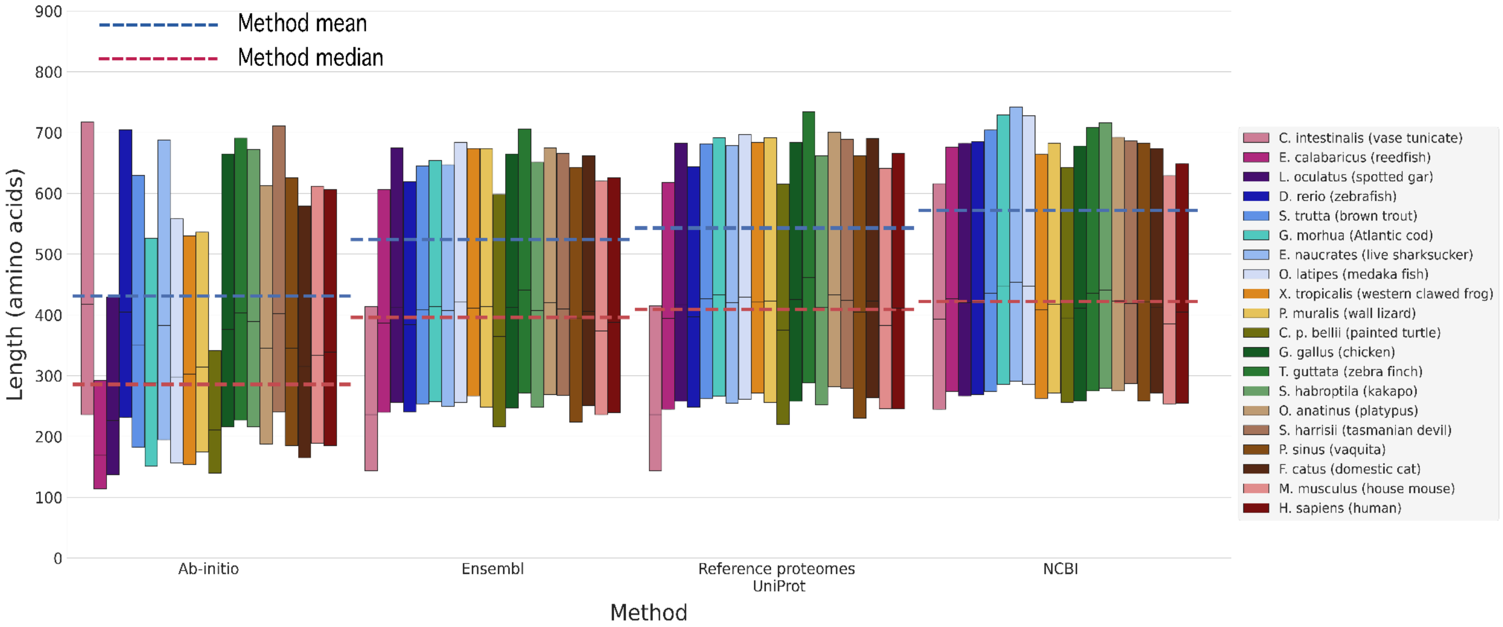

Figure S9. Protein length distribution showing all species proteomes of the four main methods. The boxplots show the median and the 0.25 and 0.75 percentiles of the distributions. The red dashed lines represent the median for the cumulative proteins of each method, and the blue dashed lines the mean for each method.

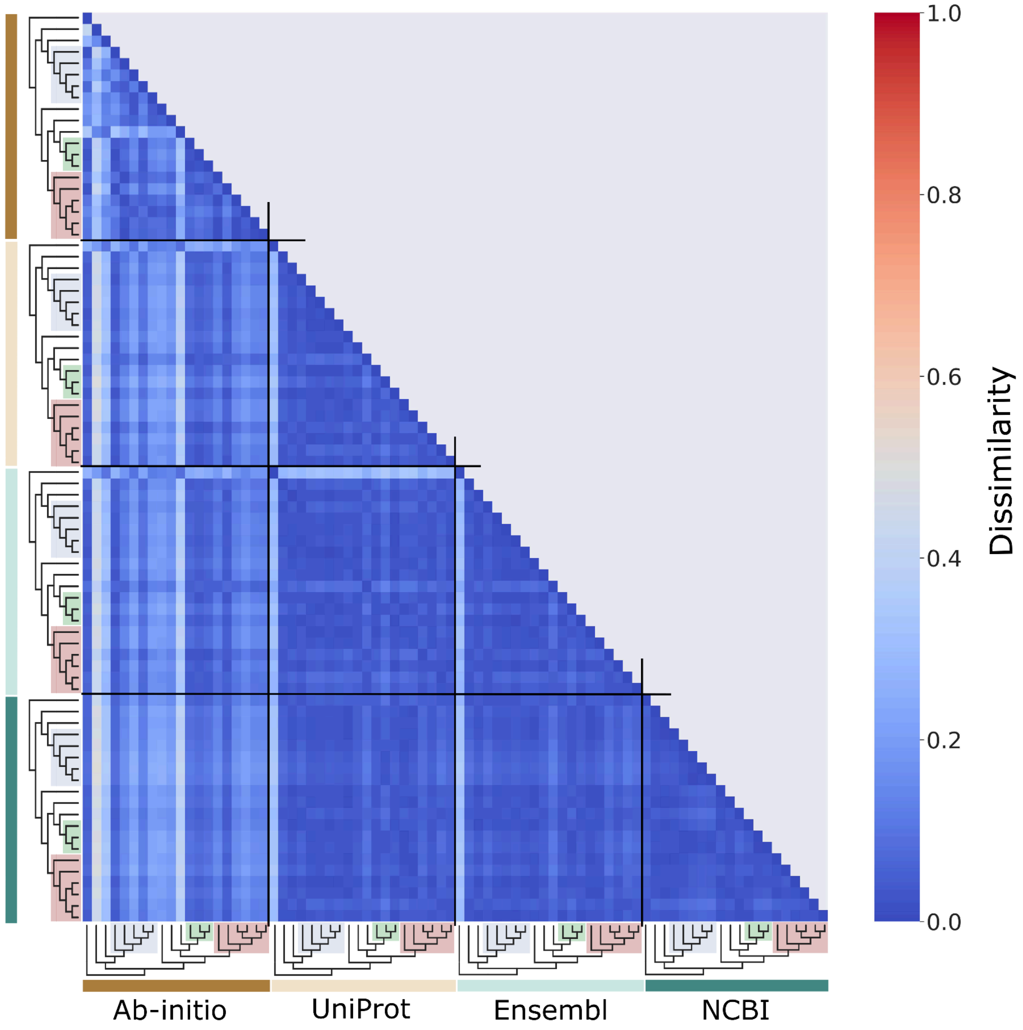

Figure S10. Pairwise dissimilarity of protein length distribution between species and methods measured by the Kolmogorov-Smirnov KS statistic.

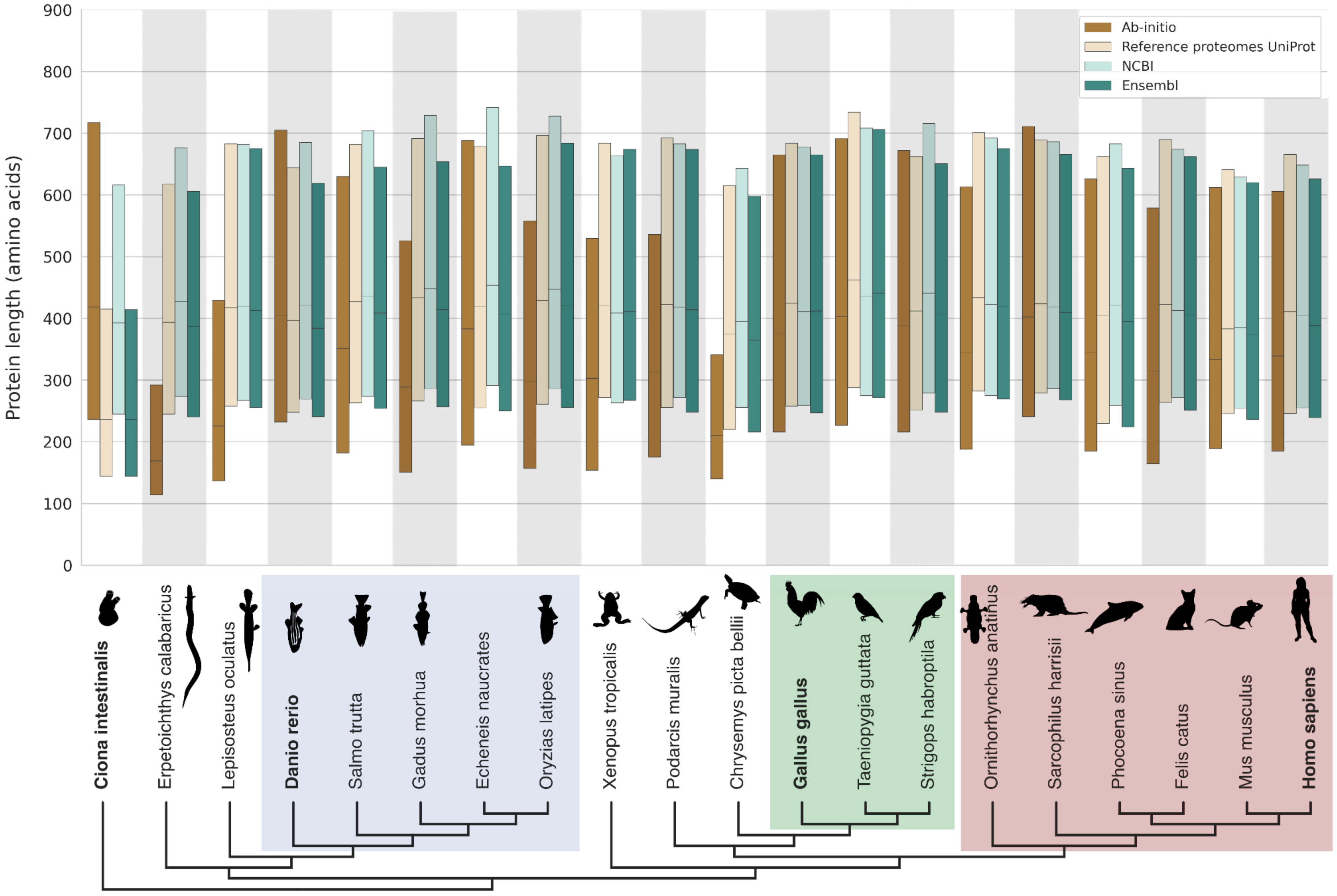

Figure S11. Gene length distribution for 4 main methods by species. For each species, the bottom of the box plot represents the first quartile, the line within represents the median, and the top line the third quartile.

BUSCO and OMArk results

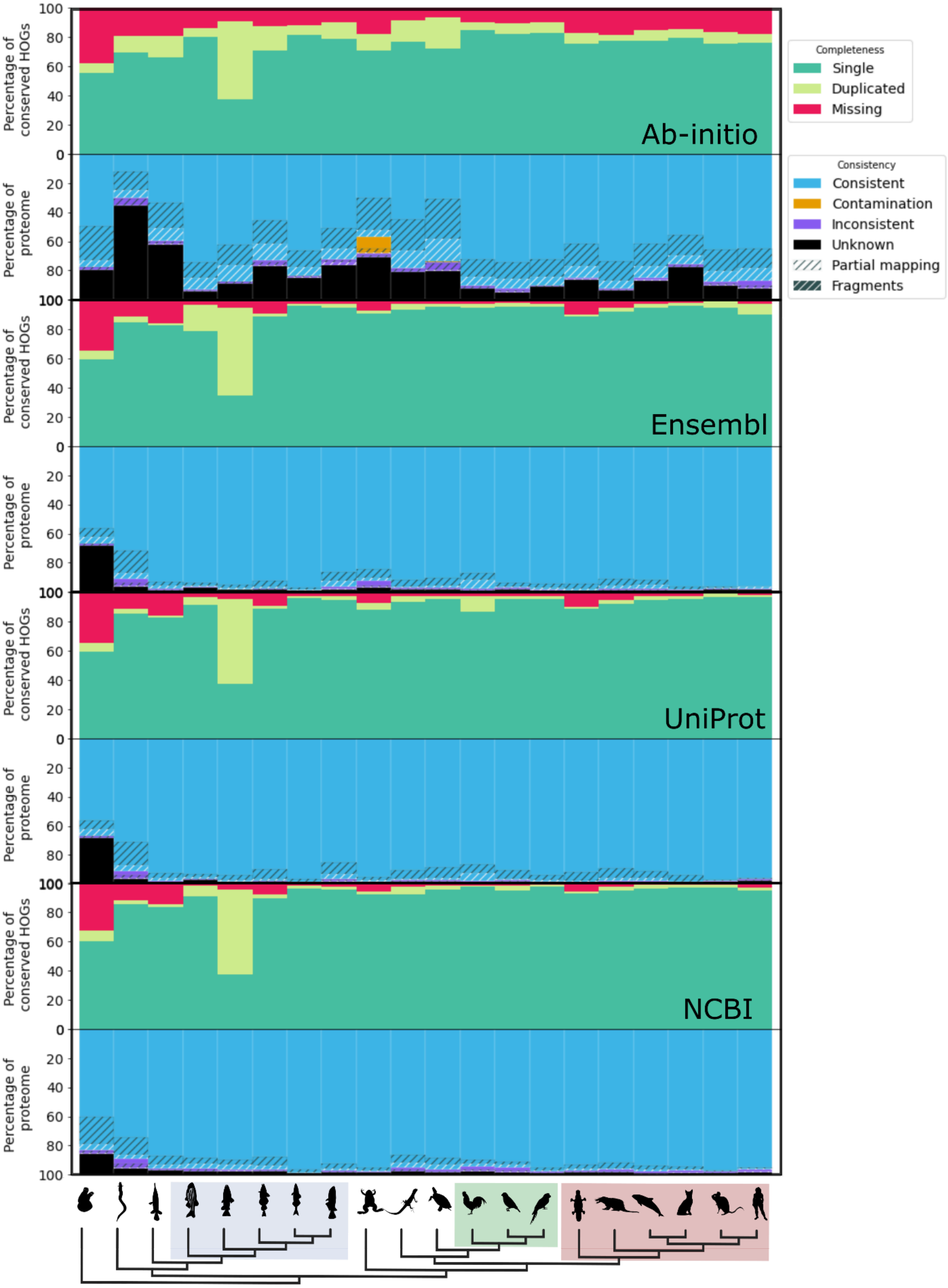

Figure S12. OMArk results for the 20 genomes and four main annotation sources in this study. The cladogram indicates the species genomes in each column of the OMArk graph.

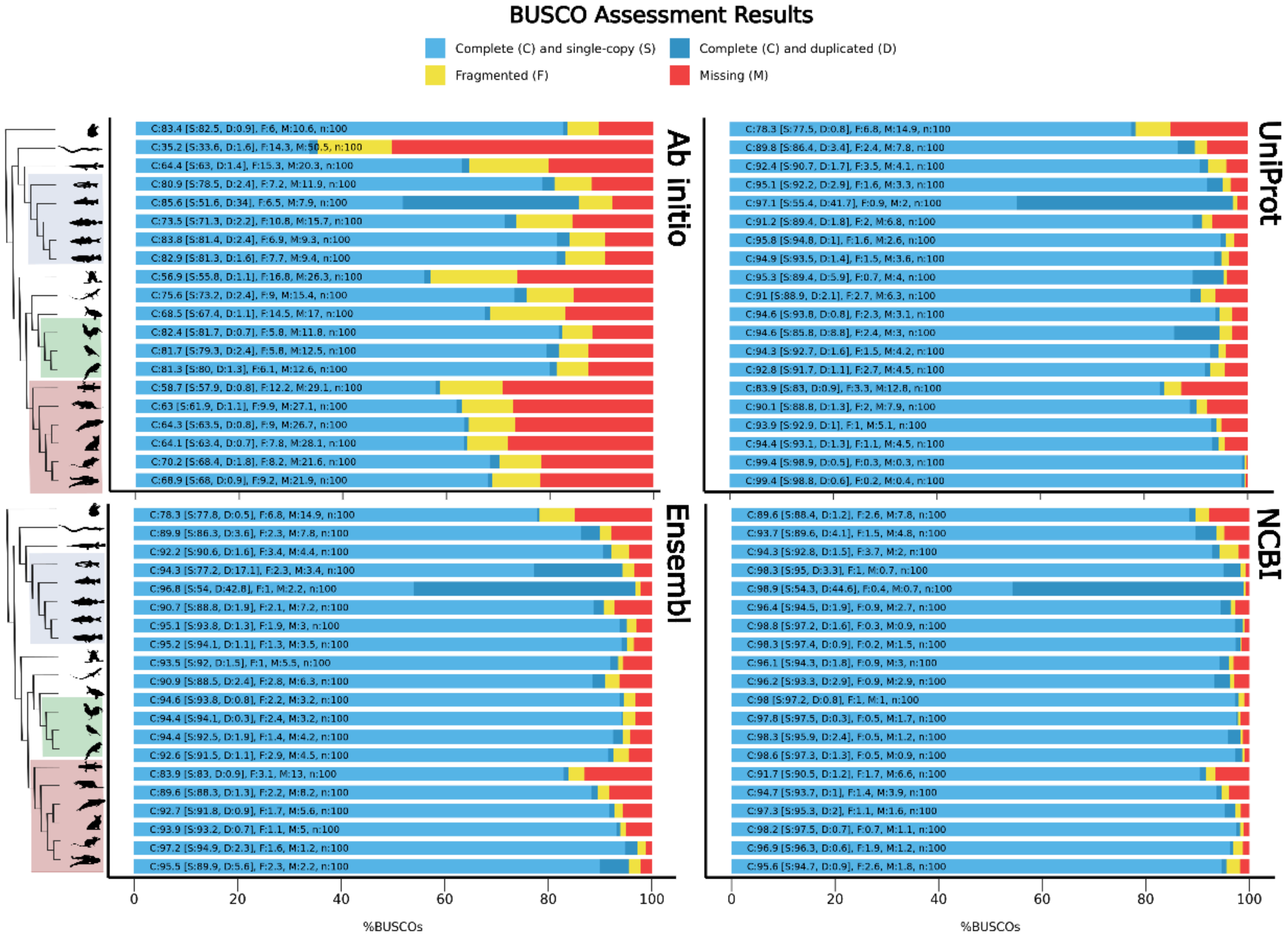

Figure S13. BUSCO results for the 20 genomes and four main annotation sources. The cladogram indicates the species genome in each row.

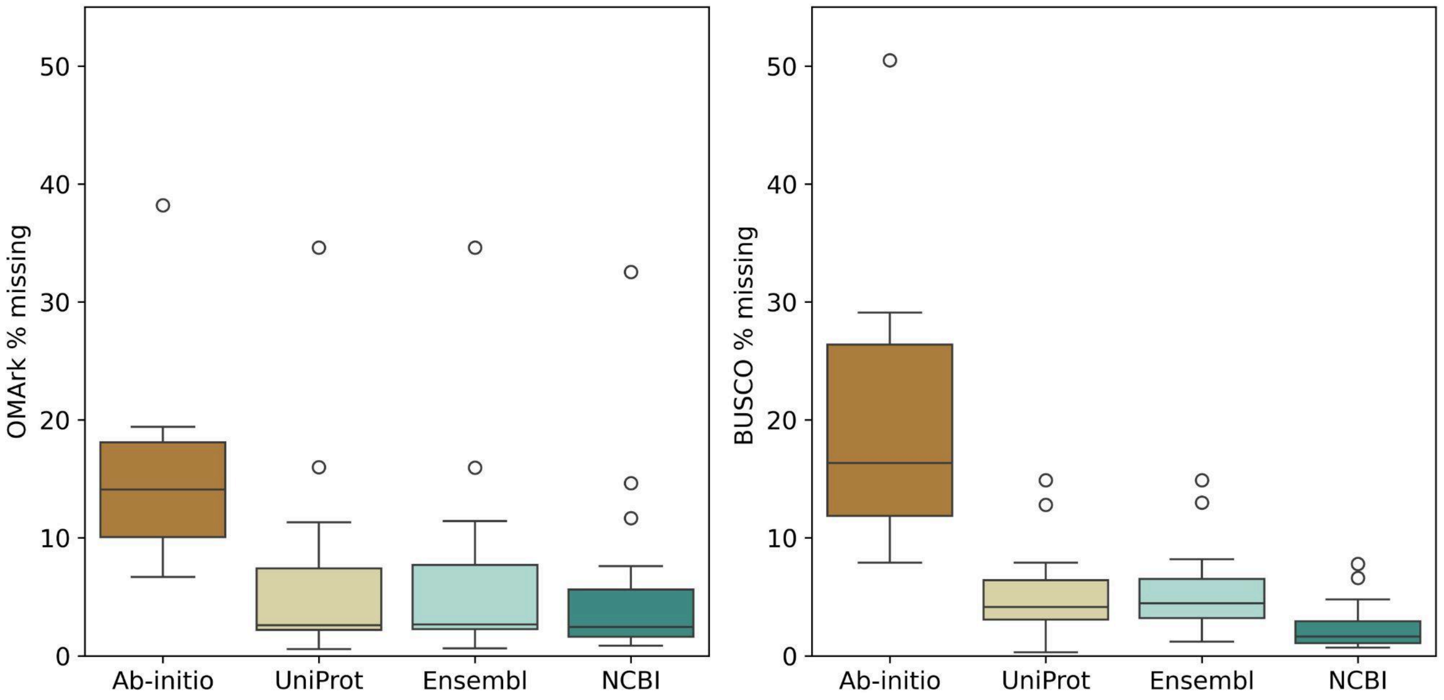

Figure S14. OMArk's (left) and BUSCO's (right) overall percent of missing genes by method.

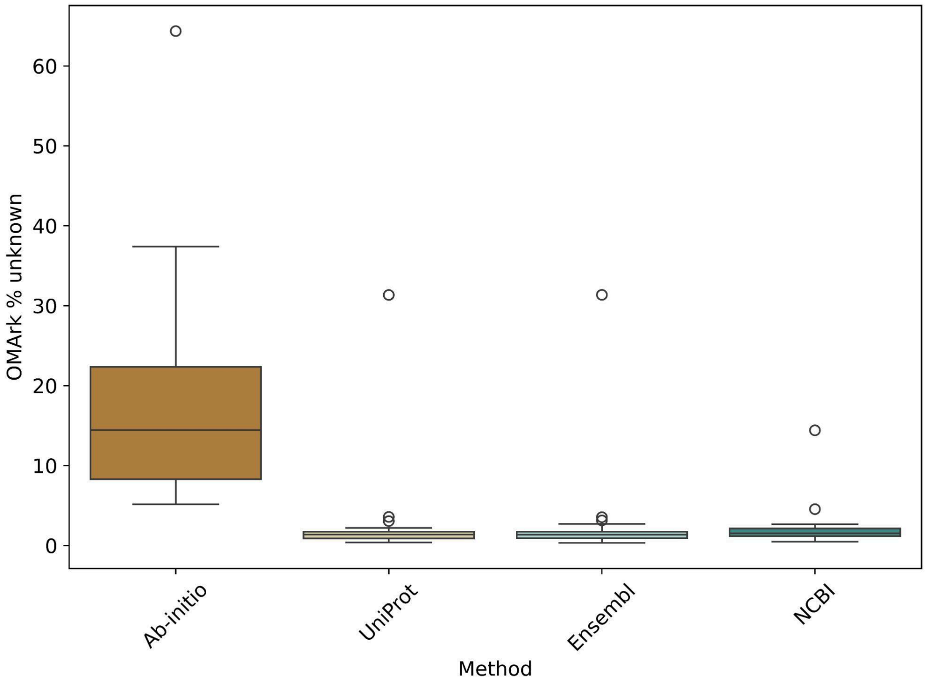

Figure S15. OMArk's overall percent of unknown genes per method.

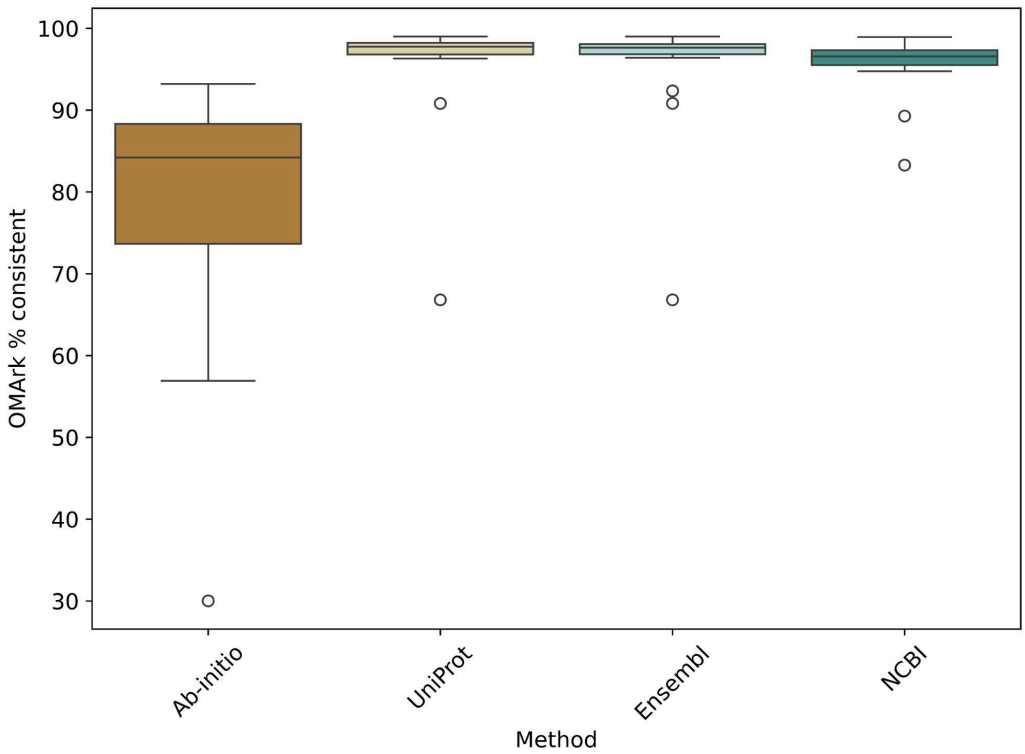

Figure S16. OMArk's percent of taxonomically consistent genes per method.

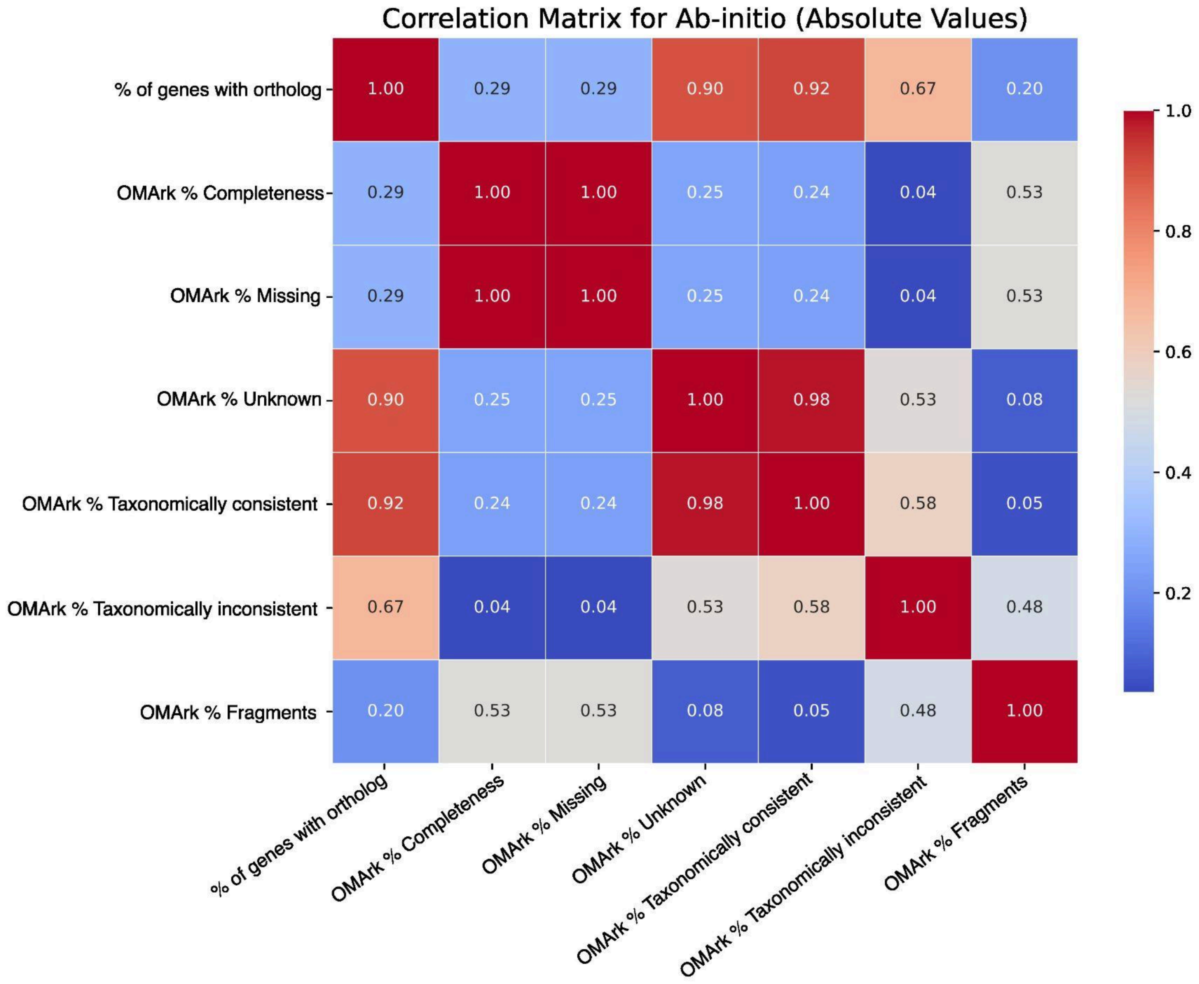

Figure S17. Correlation results between OMArk's metrics for the *ab initio* proteomes and percentage of orthologous genes.

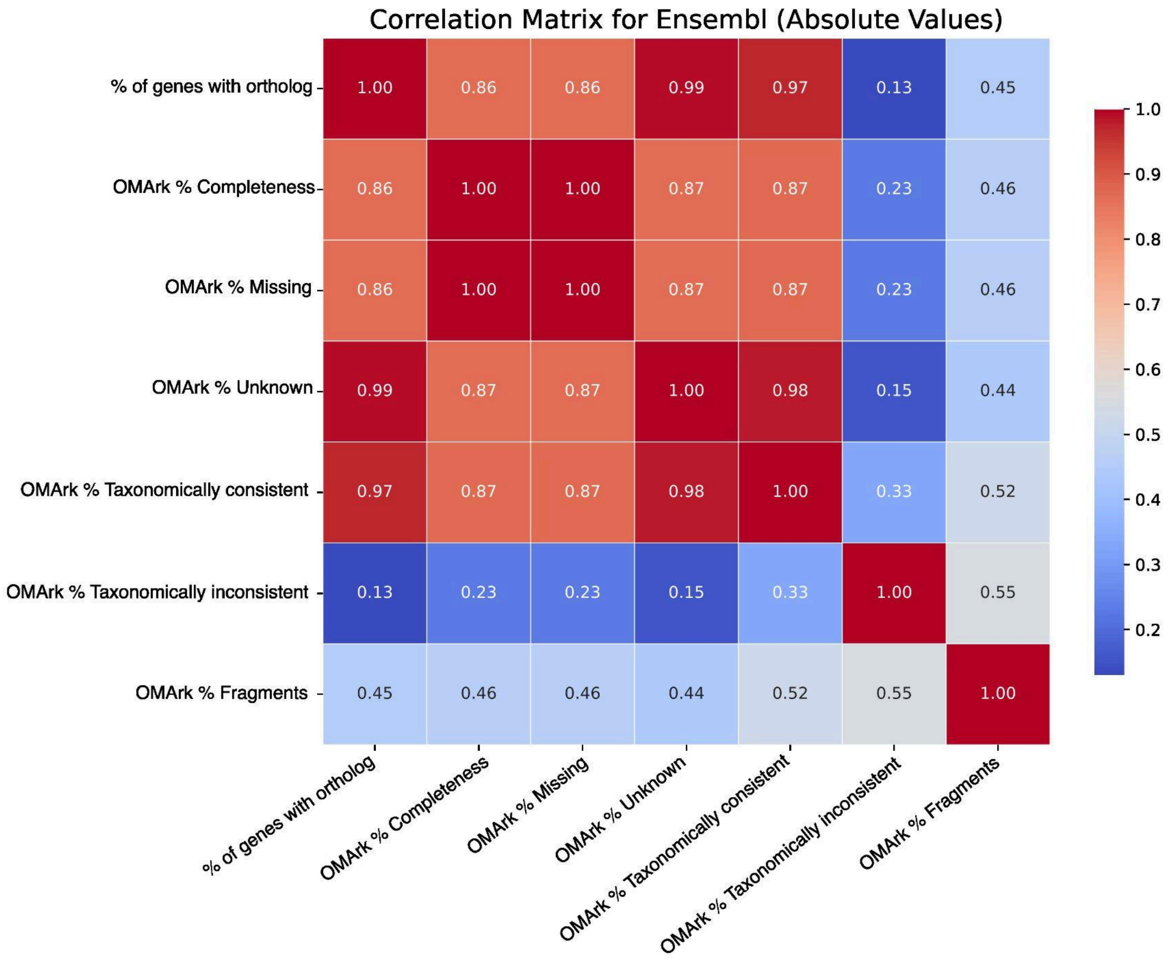

Figure S18. Correlation results between OMArk's metrics for the Ensembl proteomes and percentage of orthologous genes.

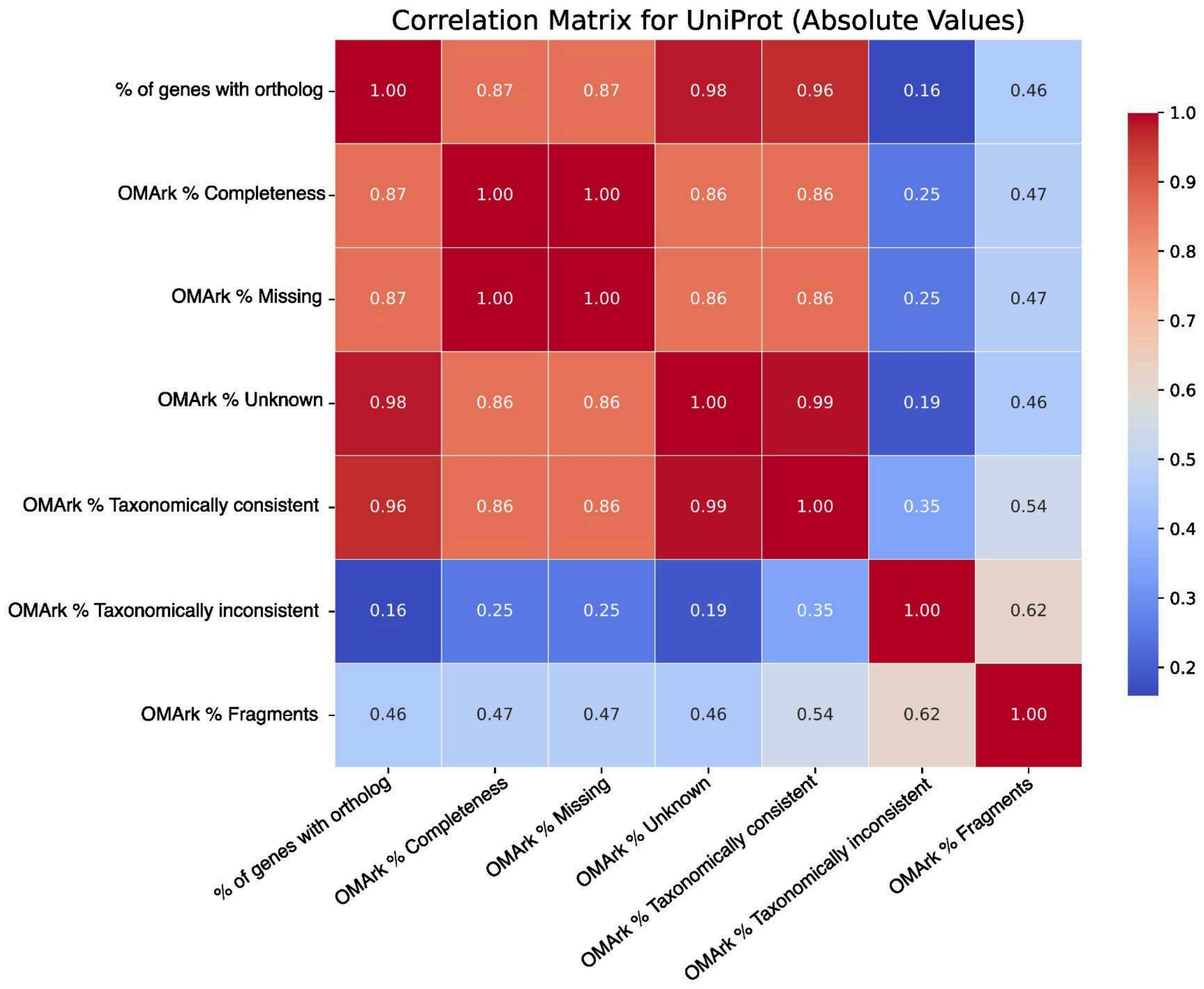

Figure S19. Correlation results between OMArk's metrics for the UniProt proteomes and percentage of orthologous genes.

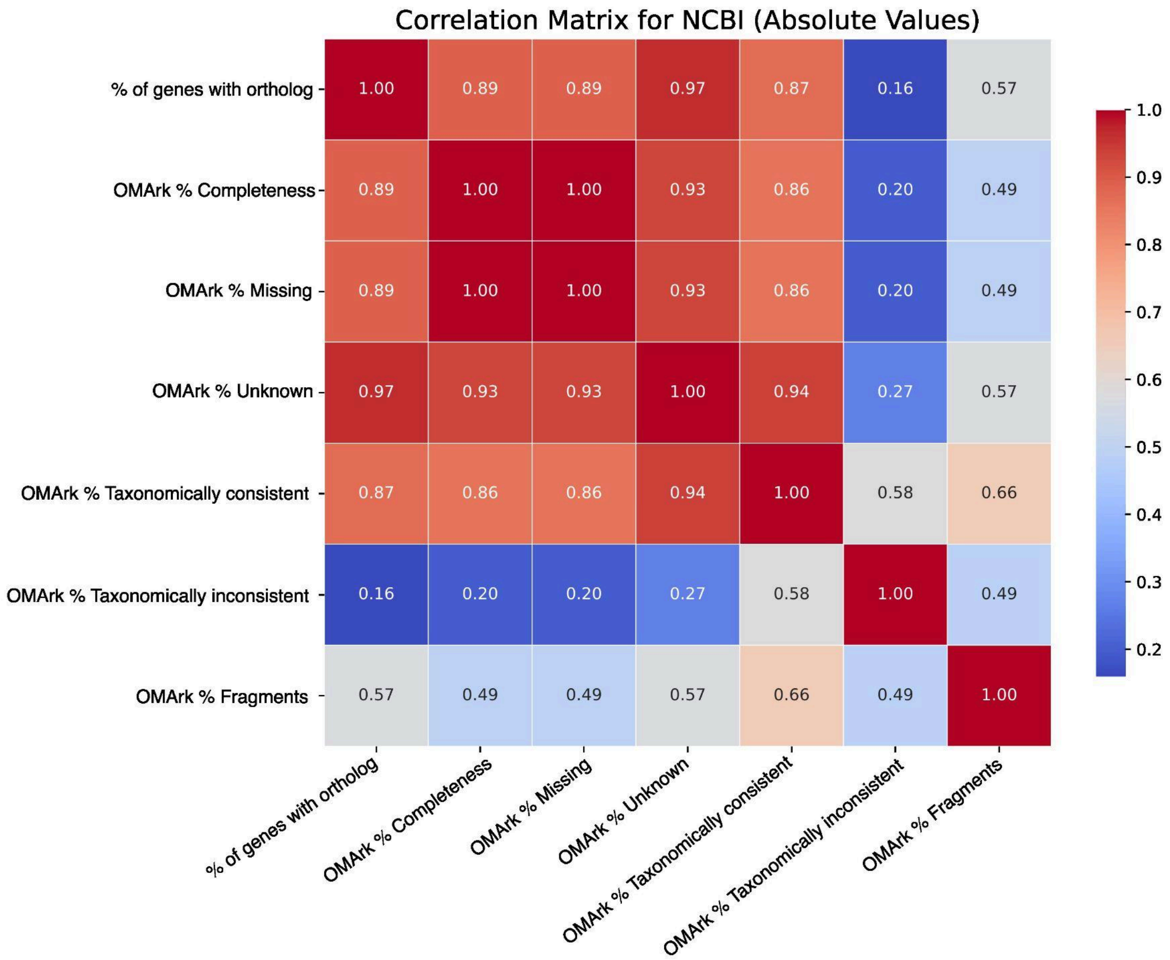

Figure S20. Correlation results between OMArk's metrics for the NCBI proteomes and percentage of orthologous genes.

Supplementary References

Altenhoff, Adrian M., Clément-Marie Train, Kimberly J. Gilbert, Ishita Mediratta, Tarcisio Mendes de Farias, David Moi, Yannis Nevers, et al. 2021. “OMA Orthology in 2021: Website Overhaul, Conserved Isoforms, Ancestral Gene Order and More.” *Nucleic Acids Research* 49 (D1): D373–79.

Casola, Claudio. 2018. “From De Novo to ‘De Nono’: The Majority of Novel Protein-Coding Genes Identified with Phylostratigraphy Are Old Genes or Recent Duplicates.” *Genome Biology and Evolution* 10 (11): 2906–18.

Emms, David M., and Steven Kelly. 2019. “OrthoFinder: Phylogenetic Orthology Inference for Comparative Genomics.” *Genome Biology* 20 (1): 238.

Kumar, Sudhir, Michael Suleski, Jack M. Craig, Adrienne E. Kasprowicz, Maxwell Sanderford, Michael Li, Glen Stecher, and S. Blair Hedges. 2022. “TimeTree 5: An Expanded Resource for Species Divergence Times.” *Molecular Biology and Evolution* 39 (8). https://doi.org/10.1093/molbev/msac174.

Vakirlis, Nikolaos, Omer Acar, Vijay Cherupally, and Anne-Ruxandra Carvunis. 2024. “Ancestral Sequence Reconstruction as a Tool to Detect and Study DE Novo Gene Emergence.” *Genome Biology and Evolution* 16 (8): evae151.

Zhang, Li, Yan Ren, Tao Yang, Guangwei Li, Jianhai Chen, Andrea R. Gschwend, Yeisoo Yu, et al. 2019. “Rapid Evolution of Protein Diversity by de Novo Origination in Oryza.” *Nature Ecology & Evolution* 3 (4): 679–90.

Zile, Karina, Christophe Dessimoz, Yannick Wurm, and Joanna Masel. 2020. “Only a Single Taxonomically Restricted Gene Family in the Drosophila Melanogaster Subgroup Can Be Identified with High Confidence.” *Genome Biology and Evolution* 12 (8): 1355–66.
